# Age-related spinal degenerative diseases: a quantitative analysis of the dynamic evolution, knowledge structure, and research frontiers in neurological damage

**DOI:** 10.3389/fneur.2026.1892694

**Published:** 2026-10-01

**Authors:** Tianyu Jiang, Shan Gao, Kaixuan Liu, Yutong Liu, Shanghe Yan, Weike Liu, Mingkun Yu

**Affiliations:** 1 Shandong Medical And Pharmaceutical University TCM Hospital, Binzhou, Shandong, China; 2 Shandong University of Traditional Chinese Medicine, Jinan, Shandong, China

**Keywords:** aging, bibliometrics, neurological outcomes, neuropathic pain, spinal degenerative diseases

## Abstract

**Background and purpose:**

Aging is the primary risk factor for the four major spinal degenerative diseases of lumbar disc herniation (LDH), cervical spondylosis (CS), lumbar spinal stenosis (LSS) and thoracic disc herniation (TDH). These diseases can lead to severe neurological dysfunction, chronic neuropathic pain and a decline in the quality of life in elderly patients. This article conducts the first simultaneous, comparative bibliometric analysis of these broader age-related spinal degenerative diseases within a unified framework, identifying the mitigation of neurological damage and neuropathic pain as the key thematic findings that currently drive the field.

**Methods:**

This study conducts a quantitative analysis of the scientific literature of LDH, CS, LSS and TDH. Our system retrieved the articles and reviews published in Web of Science, Scopus and PubMed from January 1, 2015 to April 4, 2025. We used VOSviewer and CiteSpace to draw bibliometric maps to clarify publication trends, cooperation networks and research frontiers.

**Results:**

The analysis of 15,788 documents shows that the global volume of articles is on a continuous growth trend, mainly dominated by China and the United States. Keyword co-occurrence analysis shows that “magnetic resonance imaging (MRI)” is the most core topic. The analysis of keyword burst and co-citation reveals a major paradigm shift: this field is gradually moving away from open surgery (such as laminoplasty) and traditional measurement, turning to minimally invasive neurodecompression and artificial intelligence, and paying more attention to specific pathologies such as patient-reported outcomes (PROs) and degenerative cervical myelopathy (DCM).

**Conclusion:**

This bibliometric study reveals that minimally invasive neural decompression, intelligence and precision methods are the primary research hotspots of recent progress. This field is changing from traditional surgery to a new paradigm combining endoscopic surgery, artificial intelligence decision-making and personalized elderly neuroprotection. However, readers must differentiate that these represent current academic research hotspots rather than definitively proven clinical evidence. Future research should integrate artificial intelligence, new robots, endoscopic technology and translation.

## 1 Introduction

Driven by the aging of the world’s population, LDH, CS, LSS and TDH, are highly prevalent age-related degenerative conditions of the spine, severely affecting the quality of life in elderly patients and imposing a heavy health-care and socioeconomic burden worldwide (1).

All of these conditions clinically present with pain and functional impairment. If severe, they can even lead to neurological damage, thus greatly impacting the patient’s function in the routine aspects of their lives, and their health in general. Consequently, while the overarching scientific literature encompasses a broad range of clinical and biomechanical studies, addressing and mitigating neurological outcomes has emerged as a central thematic finding within this broader context. Over the past decade, a substantial body of literature has provided deeper insights into the comprehensive clinical spectrum of four principal spinal diseases, from their underlying mechanisms and diagnostic modalities to the full range of therapeutic interventions and rehabilitation protocols (2). The scientific body on the subject is comprehensive, involving studies on molecular mechanisms, clinical trials, epidemiological investigations, and advanced imaging technologies (3). Although the individual diseases have been extensively researched, there is no analysis of the field as a whole that is comprehensive, qualitative, and visual. Specifically, there has been no systematic characterization of its collective knowledge structure, key contributors, developmental trajectory, as well. As such, although these narrative reviews have offered some qualitative insights on the literature, they are inherently limited in their objectivity and scope, falling short in synthesizing large volumes of complex data within huge bodies of literature (4). In contrast, bibliometrics, supplemented with visual analytics, provides an effective, data-driven approach to quantify the knowledge base, collaborative pattern, and developmental dynamics of a field (5). Based in a systematic analysis of scholarly metadata (authors, institutions, countries, keywords, and citations), it reconstructs the historical development of the discipline, highlights key research clusters and their impact, and paints a picture of future research promise (6). Thus, this research employs a bibliometer approach and knowledge graph visualization technique to systematically map the research landscape related to LDH, CS, of LSS and TDH in total. The literature analysis will cover global publications from the 10-year period 2015–2024 to explain the core structure, key contributors, and emerging trends of the field (7). This study tried to achieve the following goals: (1) to describe the temporal trends and the geographical distribution of the research literature; (2) to identify and evaluate the pivotal contributors to this field (like prolific authors, key institutions, and key journals) (8). To identify the thematic landscape and intellectual structure by constructing networks of keyword co-occurrence, reveal the development path of research themes, and detect emerging hotspots and trends; and to identify pivotal publications and delineate the citation networks that form the intellectual bedrock of this domain. By combining the above multi-faceted analyses, this study aimed to comprehensively describe the research landscape and intellectual structure of the knowledge domain on the four key spinal diseases. Overall, this study aims to provide an evidence-based roadmap for the scientific community, clinicians, and health policymakers. By mapping the broader literature of these four diseases, we seek to identify gaps in knowledge, promote synergistic collaborations, and guide future research—particularly highlighting the paradigm shift toward minimizing neurological damage and enhancing functional outcomes in the aging population (9, 10).

## 2 Methods

### 2.1 Data collection

We performed a systematic search for literature published between January 1, 2015, and April 4, 2025, with a limit to only retrieving original research articles and reviews. This specific 10-year window (2015–2025) was strategically selected because the mid-2010s marked a seminal historical inflection point, characterized by the global standardization of full-endoscopic spine surgeries, the breakthrough of AI in medical imaging, and the release of pivotal clinical practice guidelines (e.g., AOSpine), providing the optimal historical context for the observed paradigm shifts. In summary, our search strategy was systematically based on four major types of spinal diseases, including LDH, CS, LSS, and TDH. We used multiple search terms for each disease, which included the standard terminology, synonymous terms, and related terms. In addition, specific medical keywords (MeSH) were also used for literature retrieval in the PubMed database. In order to ensure that clear and comprehensive data sets for these four spinal diseases were obtained, separate retrievals were carried out. As an illustrative example, the query for “Lumbar Disc Herniation” was formulated as follows: “Lumbar Disc Herniation” OR “Lumbar Intervertebral Disc Displacement” OR (“Sciatica” AND “Lumbar”). The scope of all searches was restricted to the title, abstract, and keyword fields of the publications. The specific cutoff date of April 4, 2025, was selected as it represents the exact execution date of our systematic database queries, ensuring the inclusion of the most up-to-date literature available before commencing the data analysis.

To rigorously address the known issue of overlapping records across multiple databases and avoid double-counting, we implemented a two-step deduplication protocol. First, all pooled records were imported into EndNote X9 reference management software to automatically identify and merge duplicate entries based on exact matches of authors, publication years, titles, and reference types. Subsequently, CiteSpace (version 6.2.6) was employed as a secondary verification tool to remove any residual overlapping publications by strictly cross-referencing Digital Object Identifiers (DOIs) and title strings.

Following deduplication, the remaining unique records underwent a manual screening process informed by Systematic Review (SR) methodology to ensure dataset purity. As systematically detailed in our Flowchart, documents were transparently excluded based on three predefined criteria: (1) Unrelated topics (i.e., not aligned with the specified spinal degenerative diseases); (2) Irrelevant research questions (e.g., purely technical engineering papers lacking clinical or pathophysiological focus); and (3) Without full text / Reports not retrieved, which precluded comprehensive verification. For detailed search strategies, please refer to Appendix 1.

### 2.2 Bibliometric indicators

This study uses a series of core literature metrology indicators to characterize the research characteristics of this field, specifically including.

Publication Volume: This indicator represents the total number of papers for the four major spinal diseases. It is used to evaluate the productivity and activity of research at different levels (including countries/regions, institutions, authors and journals).

Citations: refers to the total number of times a paper, author or journal is cited, which is a key indicator for measuring academic influence and research importance.

Impact Factor (IF) and JCR Quartile: Mainly for the journals included in the Web of Science Core Collection (WoSCC), it is used to evaluate the academic level of the journal and its relative influence in the field of its discipline.

H-index: This is a comprehensive indicator used to evaluate the quantity and quality of academic outputs of scholars, journals, etc.

Keyword Co-occurrence Frequency and Centrality: reflects the popularity of the research topic and its core position in the knowledge network.

### 2.3 Burst detection

In order to identify emerging topics and research frontiers, this study uses the Burst Detection Algorithm in CiteSpace software to analyze the time dynamic evolution of keywords. The algorithm can identify “Burst Terms”—that is, keywords with a sharp increase in frequency during a specific period of time, and quantify their Burst Strength and the beginning and end years. This analysis clarifies the evolutionary trajectory of the research focus, highlighting the significant shift of the research focus from basic topics such as disease description and traditional treatment to emerging fields such as molecular mechanisms, new diagnostic methods and minimally invasive technologies.

### 2.4 Visualization and collaboration network analysis

This study uses VOSviewer and CiteSpace software to build and analyze a variety of knowledge networks, thus clarifying the structural characteristics and cooperation models of the research field.

Analysis of author cooperation and co-citation network: Based on the relationship of co-authorship, an author cooperation network is built to identify core research teams and influential academic communities. The author co-citation network identifies foundational or highly influential scholars and their academic schools by analyzing instances where publications jointly cite certain authors.

Journal Co-citation Network Analysis: By analyzing instances where publications jointly cite certain journals, this identifies core journals and important knowledge sources that constitute the knowledge base of the field.

Document Co-citation Network Analysis: Based on instances where documents are co-cited by other documents, this identifies classic literature, landmark studies, and key nodes in knowledge evolution within the field.

Keyword Co-occurrence Network Analysis: Based on instances where keywords appear together in the same publication, this analyzes the structure, core content, interrelationships of research themes, and the distribution of research hotspots.

### 2.5 Data analysis tools

In this study, VOSviewer (version 1.6.18) and CiteSpace (version 6.2.6) were employed for in-depth bibliometric analysis and visualization. In VOSviewer, the “full counting” method was applied. The minimum occurrence thresholds for nodes (e.g., countries, institutions, authors, and keywords) were dynamically adjusted according to specific network requirements to construct the co-occurrence and coupling maps. The clustering analysis was performed using the software’s native modularity-based algorithm. In CiteSpace, the specific parameter settings were as follows: Time Slicing was set from 2015 to 2025, with “Years Per Slice” set to 1; “Node Types” were selected according to the specific analysis targets; the “Selection Criteria” was set to Top *N* = 50. For network pruning, “Pathfinder” and “Pruning sliced networks” were applied. Keyword burst detection was conducted using Kleinberg’s algorithm with a minimum duration of 2 years.

## 3 Results

Based on the rigorous screening detailed in the Flowchart, The final data set includes 5,501 articles on LDH, 5,334 articles on LSS, 4,515 articles on CS, and 438 articles on TDH. The flow chart is as follows. As shown in Figure 1, the annual publications of these four major spinal diseases showed different development trajectories from January 1, 2015 to April 4, 2025. Research on LDH shows that the volume of publications is the highest and continues to grow significantly. More and more research on the diagnosis and treatment of LDH reflects the continuous growth in this field and special interest in the development of minimally invasive and conservative methods to manage age-related neurological pain. This reflects the high incidence of this situation. Lumbar Spinal Stenosis (LSS) publications ranked second in quantity and also showed a strong growth pattern. The volume of articles showed a steep growth curve, rising from about 340 articles in 2015 to a peak of about 675 articles in 2023. During this period, it surged in 2019 (about 525 articles), and then dropped slightly to about 650 articles in 2024. The vigorous expansion of the study of Lumbar Spinal Stenosis (LSS) reflects the increased investment by the academic community in this field. These academic inputs focus on the comparative analysis of its pathogenesis, diagnostic criteria and different treatment plans (such as the comparison of surgical and non-surgical methods and various surgical techniques). This trend may be attributed to the increased incidence of Lumbar Spinal Stenosis (LSS) and neurogenic claudication in the context of global population aging. In terms of total publications, (CS) ranks third and shows a steady growth trend, increasing from about 285 articles in 2015 to about 555 articles in 2024. During this period, the number of articles increased significantly in 2017 (about 395 articles), and two obvious peaks were formed in 2022 (about 545 articles) and 2024. The above trend shows that the academic community is paying more and more attention to cervical degenerative diseases and their related pathological changes (such as nerve root lesions and spinal cord lesions). This academic focus is highly consistent with the priority direction of clinical research, such as early diagnosis, precise intervention and functional recovery. Although the number of articles on thoracic disc herniation (TDH) is relatively small, its overall trajectory is on the rise, with significant fluctuations every year. From 2015 to 2018, the annual volume of articles remained at about 20 to 40. Starting from 2019 (about 45 articles), the number of publications began to increase, eventually reaching a peak of about 55 articles (recorded in 2021 and 2024). Although the total amount of literature on TDH is still relatively limited (which may be attributed to its low clinical incidence and technical challenges in diagnosis and treatment), its upward trend and significant peaks may reflect the relatively concentrated research investment by the academic community. These studies mainly focus on the progress of imaging diagnostic techniques (such as advanced magnetic resonance imaging sequences) and innovative treatment strategies (including various minimally invasive surgeries, such as thoracoscopy and endoscopy) to better manage this complex spinal disease.

**Figure 1:**
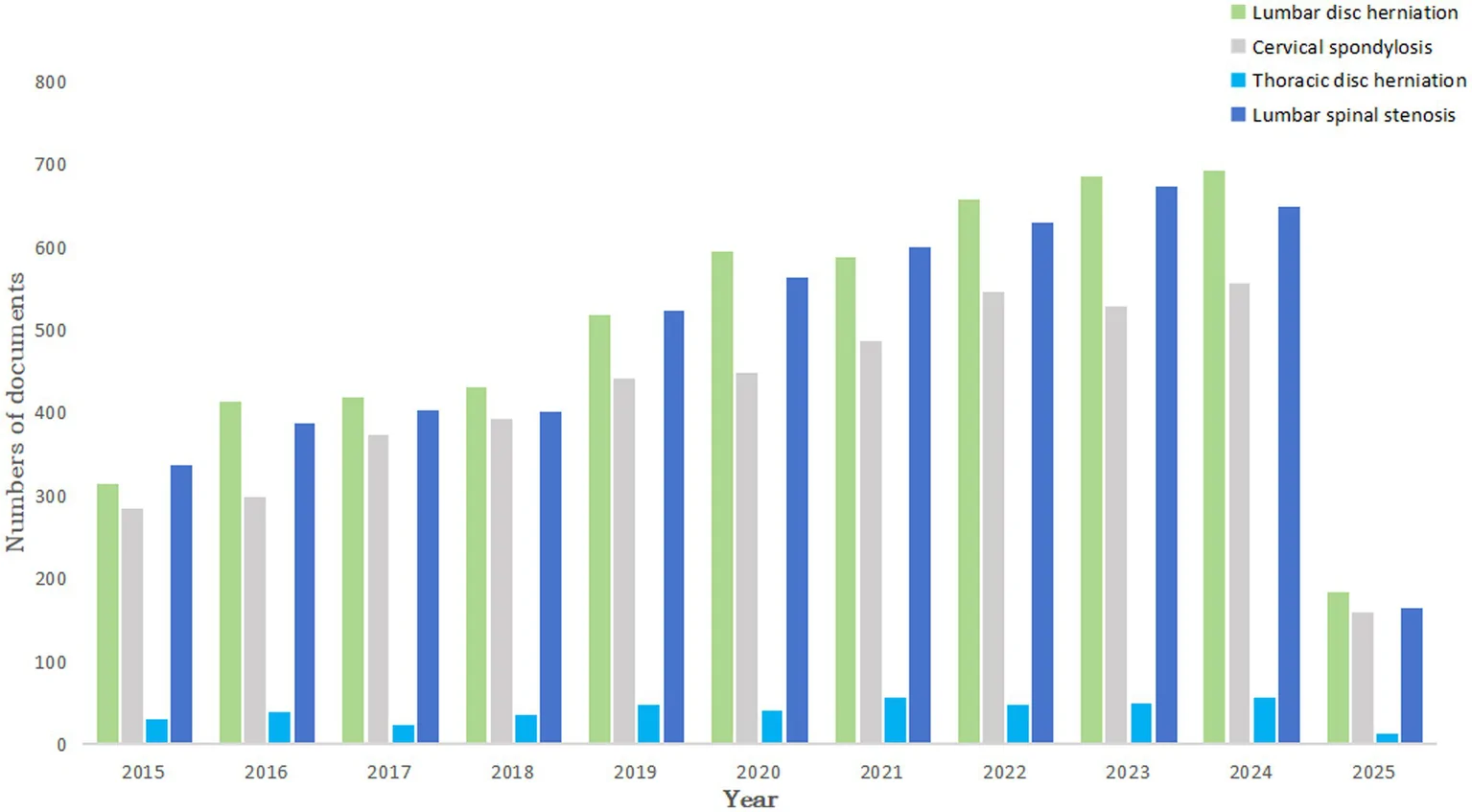
Publication trends of scientific literature on lumbar disc herniation, cervical spondylosis, thoracic disc herniation and lumbar spinal stenosis between January 1, 2015 and April 4, 2025. The data point for 2025 represents a partial publication volume up to the search cutoff date of April 4, 2025, which accounts for the apparent sharp decline on the graph.

Although the literature data for 2025 has not been fully included (as of April 4, the number of LDH articles is about 180, LSS is 165, CS is 160, and TDH is 10), the preliminary trend shows that the academic research enthusiasm in the field of spinal diseases is still continuous and strong. The distribution of research focus is significantly inclined to the field of lumbar spine lesions. The total publication volume of LDH and LSS significantly exceeds that of cervical and thoracic spine-related diseases. Looking to the future, it is expected that the research focus and growth trajectory of these different diseases will show a dynamic evolution. This evolutionary process will be shaped by multiple factors. At the clinical level, it will be affected by factors such as the unique epidemiological characteristics of each disease, advances in diagnostic techniques, and the development of rehabilitation strategies (4). At the treatment level, the paradigm shift from open surgery to minimally invasive and ultra-minimally invasive approaches will become the main driving force. In addition, emerging fields such as biomaterials and regenerative medicine are ready to develop, and are expected to open up new research frontiers.

Table 1 shows that China and the United States stand out in terms of the publication volume of the four major spinal diseases (LDH, LSS, CS and TDH) and become the most prolific regional contributors. China and the United States are the main source countries of the research literature on these four major spinal diseases (LDH, LSS, CS and TDH). As the world’s leading academic contributor, China ranks first in the number of articles published in the fields of LDH (1878 articles) and CS (1,487 articles), which reflects its extensive research investment and dedication in these common spinal lesions. Although China’s scientific research output has recently shown explosive growth (the mean publication year of LDH and CS is about 2020), the United States still dominates LSS (1,395 articles) and the relatively rare TDH (123 articles) research. A noteworthy finding is that there is a difference between the publication volume and academic impact. Although China’s number of articles in some areas exceeds that of the United States, American literature can usually accumulate higher total citations (for example, LDH with 18,775 citations; LSS with 24,252 citations), indicating that it has a more far-reaching global academic influence. At the institutional level, Capital Medical University (China) has shown excellent scientific research output and has a strong record in the fields of LDH and LSS. Many Chinese institutions, including Shandong University, Tongji University and Shanghai Jiao Tong University, have always maintained a high ranking in different disease categories, which clearly shows the depth and systematic characteristics of the country’s scientific research strength. In the United States, Harvard Medical School and its affiliated Massachusetts General Hospital have shown excellent comprehensive scientific research strength, ranking at the top of institutions in almost all four disease fields. Their leading position in the field of TDH research is particularly prominent. As world-leading medical research institutions, their outstanding influence is reflected in the high number of citations of their research results. As the two leading forces in the field of global spinal disease research, China and the United States have played a synergistic role and had a far-reaching and common impact on the evolution of this field. Although the research in this field is mainly dominated by China and the United States, a multi-center global research landscape has emerged. Other countries, including South Korea, Japan, Germany and Canada, have also made significant contributions, especially in specific disease areas or sub-disciplines.

**Table 1:** Top 10 countries/ regions and organizations related to lumbar disc herniation, lumbar spinal stenosis, cervical spondylosis, thoracic disc herniation.

Type	Rank	Country	Avg. pub. Year	Documents	Citations	Rank	Organization	Avg. pub. Year	Documents	Citations
Lumbar disc herniation	1	China	2020	1878	16,493	1	Capital Med Univ	2021	99	636
	2	USA	2019	978	18,775	2	Harvard Med Sch	2020	83	2062
	3	South Korea	2019	408	6,315	3	Shandong Univ	2020	62	813
	4	Japan	2020	253	2,704	4	Massachusetts Gen Hosp	2018	61	2060
	5	Turkey	2019	253	1886	5	Tongji Univ	2018	60	947
	6	Germany	2019	252	2,807	6	Southern Med Univ	2020	56	456
	7	India	2021	174	1,162	7	Zhejiang Univ	2020	53	430
	8	England	2019	164	3,026	8	Univ Zurich	2020	51	679
	9	Switzerland	2019	149	1962	9	Shanghai Jiao Tong Univ	2020	50	376
	10	Italy	2020	147	1,603	10	Hebei Med Univ	2020	49	344
Lumbar spinal stenosis	1	USA	2019	1,395	24,252	1	Seoul Natl Univ	2019	128	1960
	2	China	2021	1,074	9,106	2	Capital Med Univ	2021	112	767
	3	Japan	2020	692	7,618	3	Harvard Med Sch	2020	98	2056
	4	South Korea	2020	581	8,449	4	Univ Toronto	2020	83	1,546
	5	Germany	2019	252	2,846	5	Rush Univ	2020	81	1,030
	6	Canada	2019	188	4,184	6	Univ Zurich	2019	73	1,099
	7	Switzerland	2019	182	2,596	7	Seoul Natl Univ. Hosp	2018	69	1,466
	8	England	2020	150	1980	8	Yonsei Univ	2018	69	1,220
	9	Italy	2021	131	1,393	9	Mayo Clin	2020	61	2017
	10	Sweden	2020	127	2,648	10	Univ Pittsburgh	2019	57	751
Cervical spondylosis	1	China	2020	1,487	12,807	1	Univ Toronto	2019	222	8,145
	2	USA	2020	1,214	18,406	2	Univ Cambridge	2021	123	2,372
	3	Japan	2020	554	6,133	3	Univ Hlth Network	2019	108	4,137
	4	Canada	2019	315	9,918	4	Thomas Jefferson Univ	2020	89	2,434
	5	India	2020	208	1,479	5	Capital Med Univ	2021	87	580
	6	England	2020	203	3,565	6	Mayo Clin	2021	81	849
	7	South Korea	2019	196	2,185	7	Hebei Med Univ	2019	79	851
	8	Germany	2019	146	1,601	8	Univ Calif San Francisco	2021	72	1,188
	9	Switzerland	2021	121	1807	9	Second Mil Med Univ	2019	66	749
	10	Taiwan	2019	93	911	10	Univ Utah	2021	66	1,411
Thoracic disc herniation	1	USA	2020	123	1711	1	Harvard Med Sch	2020	16	430
	2	China	2020	86	665	2	Massachusetts Gen Hosp	2019	11	397
	3	Germany	2020	39	403	3	Mayo Clin	2020	8	162
	4	England	2019	32	545	4	Ohio Pain Clin	2018	8	315
	5	South Korea	2019	30	426	5	Pain Management Ctr Amer	2017	7	225
	6	Japan	2020	27	278	6	Univ Louisville	2021	7	269
	7	Italy	2021	21	61	7	Peking Univ	2021	6	73
	8	Turkey	2018	17	74	8	Univ British Columbia	2018	6	53
	9	Canada	2019	16	111	9	Univ Calif San Francisco	2020	6	63
	10	France	2020	16	167	10	Wright State Univ	2017	6	170

As shown in Figure 2A, this study uses author coupling analysis to clarify the research landscape, collaboration network and development trends in the field of lumbar disc herniation (LDH). The generated visual map highlights several prominent research clusters, among which the node size, color and connection density represent the influence of the authors and the thematic coherence of the research groups, respectively. The remarkable blue cluster centered on scholar Manchikanti, Laxmaiah is particularly noteworthy. The cluster is characterized by a large node size and dense internal connections, suggesting that this scholar has a significant academic influence in the field of interventional treatment and pain management of lumbar disc herniation (LDH), and has played a positive role in advancing this field. The research focus of this cluster may be on evaluating the efficacy of minimally invasive intervention technology and formulating relevant clinical guidelines. At the same time, a huge and dense red network mainly composed of Korean scholars such as Kim, Jin-sung and Lee, Sang-ho highlights a strong core academic group focusing on LDH surgical treatment (especially endoscopy and other minimally invasive surgeries). It can be said that the work of the research team has greatly led the evolution of LDH surgical approaches and had a profound impact on clinical standards in the Asia-Pacific and other regions. In addition, the green network composed of European and American scholars such as Claudius Thome and Alexander R. Vaccaro has also shown remarkable academic influence. The research topics of this cluster cover the following key areas: the selection of LDH surgical indications, the comparative study of traditional and new surgical techniques, and evidence-based medical evaluation of long-term efficacy and complications. The existence of these independent collaborative networks marks the diversification of LDH research dimensions. In addition, it also reflects the key process of knowledge exchange and integration between different treatment concepts (such as interventional pain management and various schools of surgery) and cross-regional academic groups. This dynamic interaction ultimately promoted the comprehensive development of research on this common spinal disease in terms of depth and breadth.

**Figure 2:**
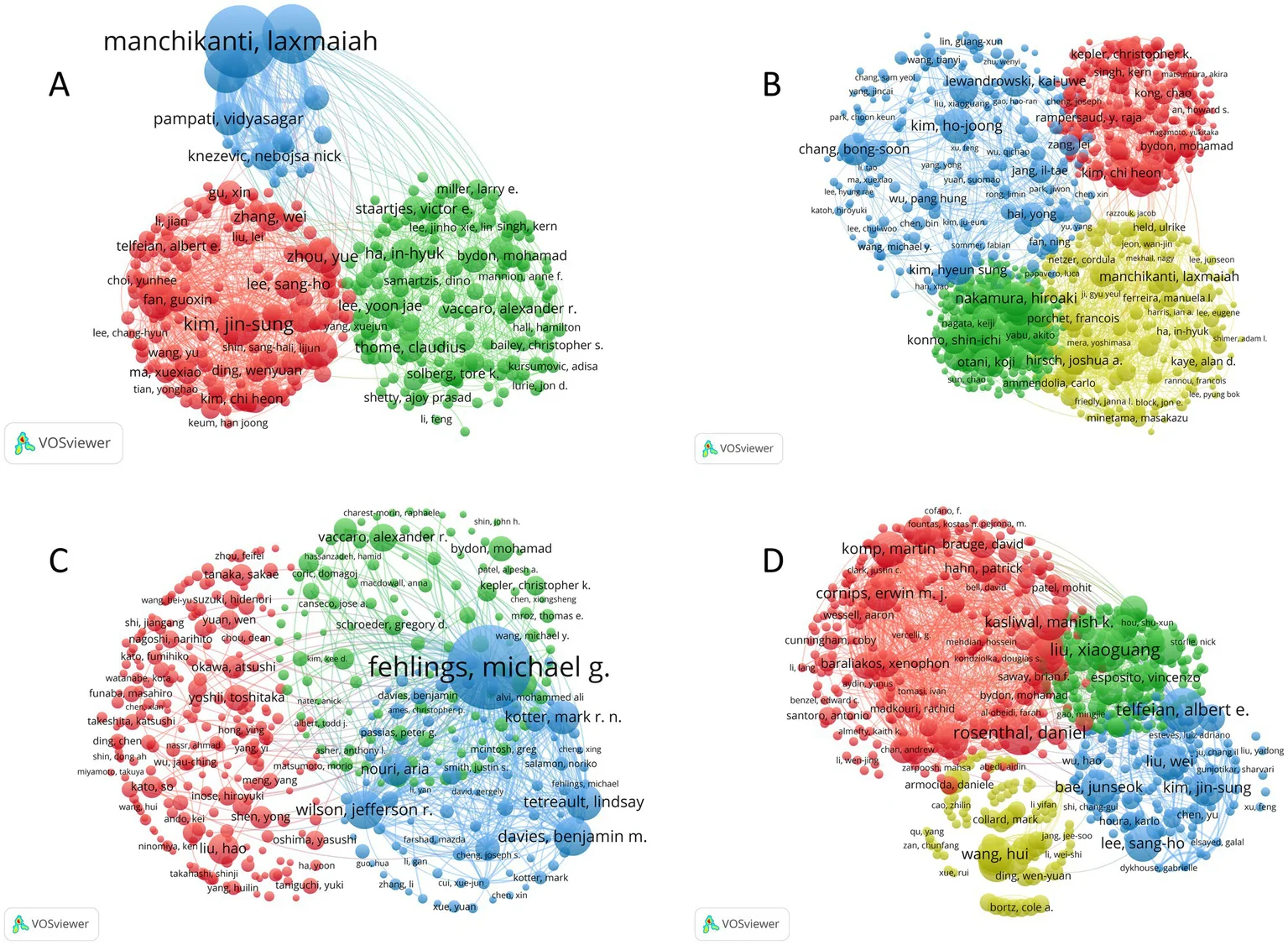
**(A)** Author coupling network for lumbar disc herniation; **(B)** Author coupling network for lumbar spinal stenosis; **(C)** Author coupling network for cervical spondylosis; **(D)** Author coupling network for thoracic disc herniation.

As shown in Figure 2B, the author coupling network of lumbar spinal stenosis (LSS) shows significant differences in structural characteristics: some fields are dominated by closely linked research clusters, while other research directions show a trend of parallel development and rarely intersect with each other. Scholar Fehlings, Michael G. stands out in an eye-catching blue research cluster and occupies a central position. His core position is intuitively reflected in the atlas as the huge node size, along with dense connections with his core collaborators (including Davies, Benjamin M. and Tetreault, Lindsay). The network structure highlights the authority and core position of the Fehlings team in many key areas of LSS research (covering neurofunction assessment, surgical decision-making, prognosis prediction and translational research). It can be considered that their research has laid a solid theoretical foundation for precision treatment and neuroprotection strategies, especially related to the recovery of nerve plasticity and function after decompression of the aging spinal cord, and provided key clinical evidence. A Japanese research team led by scholars such as Yoshii, Toshitaka and Okawa, Atsushi, constitutes another important research cluster, which is displayed as a red network in the map. As a key force in the field of LSS surgical technology, the team’s research may focus on treatment strategies and clinical outcomes tailored to populations in Japan and East Asia, which may include in-depth analysis of specific surgical approaches and fusion techniques. In addition, the existence of smaller clusters represented by the green network is also worthy of attention. These clusters are likely to represent an in-depth exploration of specific niche areas, such as distinct LSS pathologies (such as spinal canal stenosis combined with degenerative lumbar spondylolisthesis) or dynamic stabilization and other new treatment concepts. Overall, these diversified collaborative networks reflect the multidimensional characteristics of LSS research and promote academic collaboration in order to explore the best treatment solutions for this complex degenerative disease.

The research field of (CS; Figure 2C) reflects a multi-center, specialized collaborative model similar to lumbar spinal stenosis (LSS), which is characterized by the formation of a conspicuous blue network centered on Kim, Ho-joong and Chang, Bong-soon, which is manifested by large node sizes and tight internal connections. The network structure highlights the core role and academic influence of the team in the field of CS (cervical spondylosis) surgical treatment. Its research expertise mainly focuses on the application of advanced surgical techniques (such as cervical artificial disc replacement and fusion) and the rigorous evaluation of clinical efficacy. The team’s research results are expected to provide important guidance not only for South Korea, but also for the clinical practice of international cervical spine surgery. At the same time, a green important network led by Japanese scholars such as Nakamura, Hiroaki and Konno, Shin-ichi has become another key research force in this field. The main research interests of this cluster focus on the field of (CS), especially ossification of the posterior longitudinal ligament (OPLL) and other special subtypes of diseases. Its research scope includes epidemiology, underlying pathological mechanisms and various treatments. It is worth noting that Laxmaiah Manchikanti once again became the core of the unique yellow star group. The cluster is connected to the blue and green networks that focus on surgical treatment. This structural connection highlights the important position of interventional pain management in the comprehensive treatment of (CS). In addition, it shows that there is a common literature basis and potential clinical intersections between this research direction and the field of surgery. The internal cohesion of these networks provides strong evidence for the dynamic interaction between different treatment concepts (such as surgical treatment and interventional treatment) and different geographical research priorities. It is this academic synergy that ultimately promotes the continuous expansion of the depth and breadth of (CS) research.

Although the number of articles on thoracic disc herniation (TDH) is significantly lower than the number of articles on lumbar and cervical spine diseases, the author-coupled network shown in Figure 2D still effectively outlines the core research force and cooperation model in this highly specialized field. Among them, the yellow network centered on scholar Rosenthal, Daniel is particularly eye-catching because of its huge node size and tight internal connections. These visual features clearly demonstrate his authoritative position and far-reaching influence in the field of TDH surgical treatment, especially in minimally invasive surgical approaches such as thoracoscopy. It can be assumed that the team’s research mainly focuses on three key areas: improving TDH surgical methods, evaluating the therapeutic efficacy, and formulating complications management strategies. At the same time, a prominent red cluster mainly composed of European scholars such as Komp, Martin and Cornips, Erwin M. J., also appeared. The network represents an active research force, especially committed to the promotion of ultra-minimally invasive technologies of TDH, such as transforaminal endoscopic surgery. Green clusters, represented by Liu Xiaoguang and other well-known Chinese scholars, reflect the growing influence of Asian research in the field of TDH. The cluster, together with other identified networks, shows that even in the field involving relatively rare pathologies, a clear model of global knowledge dissemination and international cooperation has been formed. This cooperative scientific research plays a crucial role in overcoming technical challenges and optimizing treatment strategies. Ultimately, it will help deepen the understanding of this complex and challenging spinal disease and improve its clinical management.

This study systematically identifies the knowledge base and core academic contributors of the four major spinal diseases (lumbar disc herniation, lumbar spinal stenosis, cervical spondylosis and thoracic disc herniation). The detailed results are shown in Figures 3A–D and Table 2.

**Figure 3:**
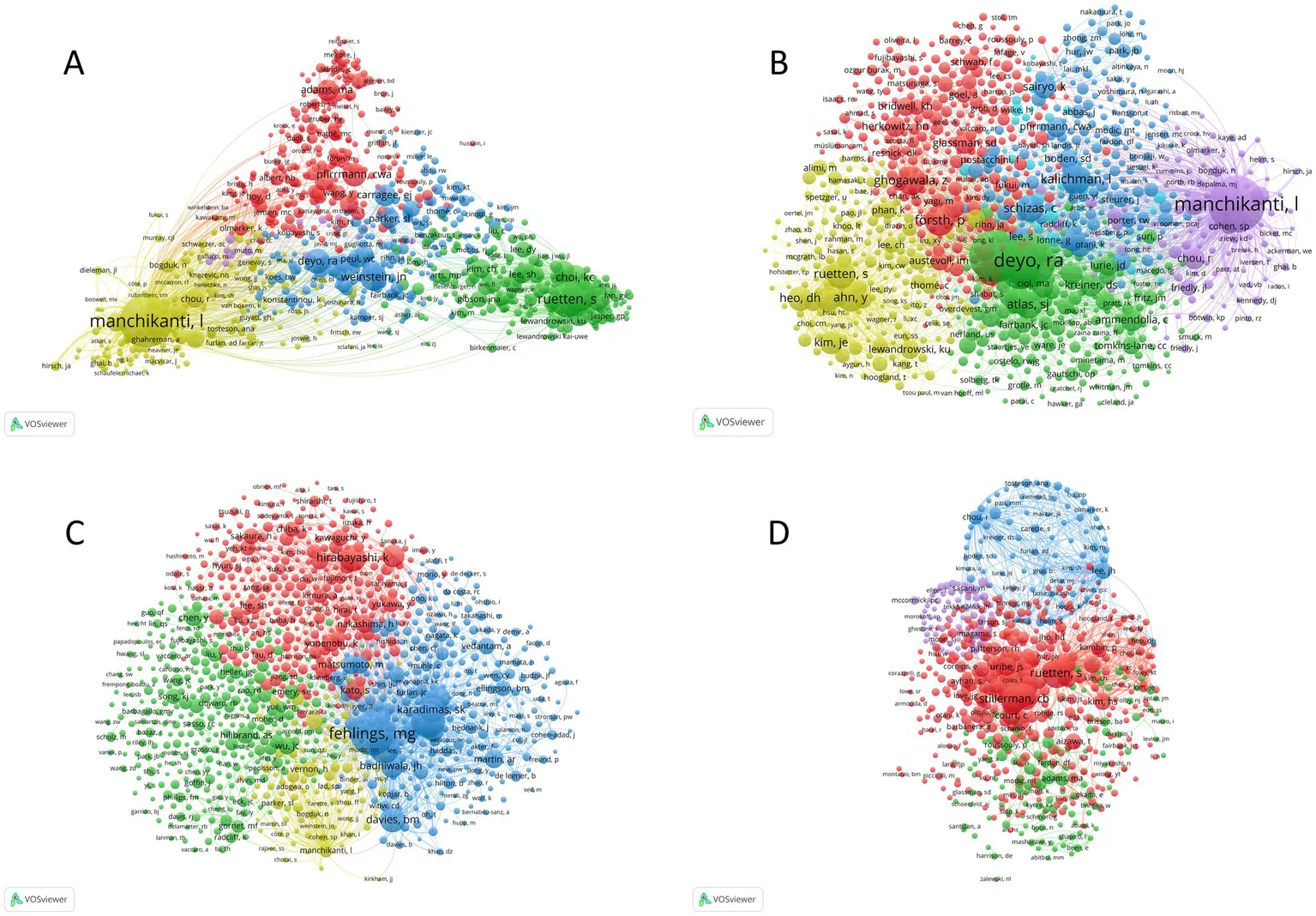
**(A)** Author co-citation network for lumbar disc herniation; **(B)** Author co-citation network for lumbar spinal stenosis; **(C)** Author co-citation network for cervical spondylosis; **(D)** Author co-citation network for thoracic disc herniation.

**Table 2:** Top 10 authors and co-cited authors related to lumbar disc herniation, lumbar spinal stenosis, cervical spondylosis, thoracic disc herniation.

Type	Rank	Author	Avg. pub. Year	Documents	Citations	Rank	Co-cited author	Citations
Lumbar disc herniation	1	Manchikanti, L	2018	65	2,366	1	Manchikanti, L	2,694
	2	Hirsch, Joshua A.	2018	52	1,696	2	Ruetten, S	1,201
	3	Kaye, Alan D.	2019	37	1,418	3	Ahn, Y	1,044
	4	Kim, Jin-Sung	2020	36	773	4	Weinstein, JN	701
	5	Ahn, Yong	2020	31	695	5	Choi, KC	645
	6	Lewandrowski, Kai-Uwe	2020	29	343	6	Deyo, RA	604
	7	Pampati, Vidyasagar	2019	27	874	7	Pfirrmann, CWA	549
	8	Zhou, Yue	2018	27	618	8	Kambin, P	542
	9	Ha, In-Hyuk	2020	24	177	9	Yeung, AT	504
	10	Lee, Sang-Ho	2018	23	422	10	Choi, G	495
Lumbar spinal stenosis	1	Kim, Ho-Joong	2019	41	936	1	Manchikanti, L	1,457
	2	Nakamura, Hiroaki	2020	40	394	2	Deyo, RA	1,324
	3	Chang, Bong-Soon	2018	35	852	3	Weinstein, JN	1,253
	4	Lewandrowski, Kai-Uwe	2020	34	384	4	Katz, JN	805
	5	Manchikanti, L	2018	34	1,148	5	Försth, P	483
	6	Hermansen, Erland	2022	33	521	6	Kalichman, L	464
	7	Indrekvam, Kari	2022	33	521	7	Ruetten, S	456
	8	Brisby, Helena	2022	32	360	8	Ghogawala, Z	452
	9	Hellum, Christian	2021	32	531	9	Ahn, Y	428
	10	Kim, Hyeun Sung	2020	32	759	10	Atlas, SJ	425
Cervical spondylosis	1	Fehlings, MG.	2019	192	7,438	1	Fehlings, MG	1756
	2	Davies, BM.	2021	71	1,272	2	Nouri, A	1,088
	3	Wilson, Jefferson R.	2020	71	2,104	3	Tetreault, L	881
	4	Tetreault, Lindsay	2018	66	3,758	4	Hirabayashi, K	672
	5	Kotter, Mark R. N.	2020	60	1,432	5	Davies, BM	589
	6	Nouri, Aria	2018	56	2,716	6	Karadimas, SK	578
	7	Liu, Hao	2020	53	413	7	Tetreault, LA	528
	8	Vaccaro, Alexander R.	2020	47	614	8	Goel, A	502
	9	Yoshii, Toshitaka	2020	42	555	9	Machino, M	484
	10	Bydon, Mohamad	2021	41	417	10	Badhiwala, JH	466
Thoracic disc herniation	1	Manchikanti, L	2019	12	481	1	Manchikanti, L	528
	2	Hirsch, Joshua A.	2019	11	406	2	Ruetten, S	102
	3	Soin, Amol	2019	9	367	3	Stillerman, CB	87
	4	Kaye, Alan D.	2020	8	304	4	Yoshihara, H	69
	5	Pampati, Vidyasagar	2020	8	235	5	Manchikanti, L	67
	6	Sanapati, Mahendra R.	2021	6	150	6	Hott, JS	61
	7	Komp, Martin	2019	4	100	7	Quint, U	58
	8	Ruetten, Sebastian	2019	4	100	8	Arts, MP	55
	9	Telfeian, Albert E.	2020	5	98	9	Ahn, Y	53
	10	Lee, Sang-Ho	2019	4	71	10	Schievink, Wi	52

As shown in the citation network of authors in Figure 3A and Table 2, the research field of lumbar disc herniation (LDH) is based on a mature and highly structured knowledge system. Unlike the research field driven by a single methodology or a broad multidisciplinary model, the research panorama of LDH is defined by the dominant cluster of experts focusing on different clinical treatment paths and basic scientific principles. Clinical efficacy and technical optimization have become the fundamental driving force for shaping the production of knowledge in this field. Therefore, the study panorama is condensed into two main clusters, and each cluster represents a dominant treatment paradigm. As a representative of one of the main treatment models, the yellow cluster on interventional pain management relies on the academic contribution of Manchikanti, L.; as the central node of the cluster, he obtained up to 2,694 significant co-citation frequencies. The academic contributions of this research group have greatly promoted the development of evidence-based guidelines for non-surgical treatments. Well-known scholars in the cluster, including Hirsch, J. A. (1,696 co-citations), have provided rigorous evidence support for epidural steroid injections and other techniques. Innovation in the field of minimally invasive surgery is mainly driven by the green cluster. The group is led by outstanding spinal endoscopy experts, including S. Ruetten, who has garnered 1,201 co-citations, and G. Choi. The surgical treatment of lumbar disc herniation (LDH) has evolved towards less traumatic and faster -recovering procedures, which is largely driven by this research group. Their research focuses on promoting the application of endoscopic approaches, conducting comparative effectiveness research against traditional surgery, and formulating strategies to minimize complications. Two basic clusters provide support for these clinical applications, among which the blue cluster, represented by J. N. Weinstein (701 co-citations) and R. A. Deyo, constitutes the cornerstone of clinical evidence-based decision-making. High-level evidence on the natural history of LDH and the long-term efficacy of various treatments is mainly established through its landmark clinical trials (such as SPORT research). An important methodological contribution of these trials is that they strongly advocate the application of patient-reported outcomes (PROs). The basic research on the pathogenesis of lumbar disc herniation (LDH) is mainly reflected in the red cluster, which relies on M. A. Adams (580 co-citations) and C. W. A. Pfirrmann. Through in-depth exploration of basic fields such as intervertebral disc biomechanics, cell degeneration and standardized imaging evaluation (such as Pfirrmann grading), the research of this group provides crucial theoretical support for understanding the nature of the disease and developing new treatment methods. As revealed by the co-citation network, the essential feature of the LDH research field lies in its professional academic community structure, where each community is driven by the influence of leading scholars. This configuration fully confirms that LDH research has formed a mature and comprehensive system. In this system, the parallel optimization of different treatment modalities (interventional and surgical), coupled with a deep, evidence-based understanding of the underlying pathology and clinical course of the disease, has jointly promoted the continuous evolution of knowledge in this field.

A key difference from LDH is that the intellectual base of LSS (lumbar spinal stenosis) research, revealed by the author co-citation network (Figure 3B; Table 2), focuses more on the principles of evidence-based medicine. Epidemiological and large-scale clinical trial research is mainly dominated by the green cluster, which is led by R. A. Deyo (750 co-citations) and S. J. Atlas, alongside other outstanding scholars. As a supplement, the blue cluster, represented by L. Kalichman (480 co-citations) and S. D. Boden, is committed to exploring the pathophysiological basis of LSS (such as ligamentum flavum hypertrophy) and imaging standards. By providing an indispensable diagnostic and etiological basis for the development of precise diagnosis and non-surgical treatment, this research has paved the way for the emergence of several treatment-oriented, independent research fields. The purple cluster, with Manchikanti, L., as its absolute core (2,350 co-citations), represents the authoritative voice in the field of interventional pain management, providing a key evidence base for techniques such as epidural steroid injections. The red cluster, represented by S. D. Glassman (620 co-citations) and K. H. Bridwell (590 co-citations) and other spinal surgery authorities, is centered on addressing the core problems of LSS surgical treatment, covering the selection of decompression and fusion strategies, technical optimization, and the evaluation of long-term clinical outcomes. The smaller yellow cluster centered on S. Ruetten (350 co-citations in total) highlights the growing attention of the academic community to ultra-minimally invasive technology. The cluster study seems to focus on the endoscopic surgical approaches of a specific LSS subtype. In short, the knowledge structure in the field of LSS is based on the core pillars of evidence-based medicine and epidemiology. Various independent but interrelated research branches—covering intervention, surgery and diagnostic methods-draw their guiding principles and evidence from this pillar.

The author co-citation network of (CS; Figure 3C) reveals a more complex and highly subdivided knowledge panorama than similar lumbar diseases. A key feature of this field is the emergence of highly specialized research clusters. These academic groups mainly gather around two main axes: specific pathological subtypes—the most prominent of which is cervical spondylotic myelopathy (CSM)—and different surgical concepts. M. G. Fehlings (1,756 co-citations) and his core collaborators (including J. R. Wilson, L. Tetreault and A. Nouri) lead and dominate the blue cluster, representing the authoritative academic system in the field of CSM and related spinal cord injuries. Through pioneering large-scale prospective research and cutting-edge translational research (such as the AOSpine CSM series), the team defined the core clinical dimensions of CSM (covering all aspects from pathophysiology to prognostic factors) and led the development of evidence-based medicine and clinical guidelines for this complex disease. This analysis also identifies two completely different schools of surgery: the red cluster centered on K. Hirabayashi (672 co-citations) represents the classic posterior cervical approach, which is typically represented by Hirabayashi open-door laminoplasty. The research group is mainly composed of East Asian scholars, who are committed to improving the posterior surgical techniques and evaluating its long-term efficacy. Conversely, the green cluster anchored by A. R. Vaccaro (614 co-citations) and A. Goel has laid the foundation for anterior cervical procedures (including ACDF and ACCF). In a word, the analysis shows that the knowledge panorama of CS is supported by three core research pillars: CSM comprehensive evidence-based research led by the Fehlings team, the posterior surgical school represented by Hirabayashi, and the anterior surgical school represented by Vaccaro.

As shown in Figure 3D and Table 2, the author co-citation network of thoracic disc herniation (TDH) presents a unique structure, which can be attributed to the lower prevalence of the disease. Compared with more common spinal diseases, the intellectual landscape of TDH is highly concentrated and is mainly shaped by the accumulated experience and technological innovation of a few core expert groups focusing on high-risk surgeries and diagnostics. The red cluster, anchored by C. B. Stillerman (87 co-citations) and H. Yoshihara alongside other highly co-cited authors, represents the basic literature on traditional open surgery, with techniques such as the transthoracic approach as the typical representative. Although a major research field is committed to building an existing knowledge system to deal with complex TDH and its related complications, the second largest research field, led by S. Ruetten and other scholars, marks a shift to the paradigm of minimally invasive spinal surgery. In order to establish a reliable alternative to traditional open surgery, the research in this cluster is committed to the development and clinical verification of a series of minimally invasive technologies, and highly emphasizes the application of endoscopic technology. The third, more dispersed blue cluster further enriches this research landscape. This cluster focuses on diagnostics and pathophysiology, aiming to provide scientific support for the two dominant surgical schools. Core topics, such as establishing advanced imaging standards for calcified or giant TDH, are highly dependent on the research results of neurosurgical experts in the cluster, among which W. I. Schievink (52 co-citations) is the most prominent. Research in this field plays an important role in improving diagnostic accuracy and building a solid theoretical framework to guide clinical decision-making. In summary, the knowledge structure in the field of TDH is characterized by the binary axis of technical expertise. This axis is shaped by the coexistence of two completely different surgical concepts: the inheritance of complex open surgery and the rise of innovative minimally invasive technology.

In order to clarify the knowledge base in this field, this study conducted a journal coupling analysis of lumbar disc herniation (LDH; Figure 4A; Supplementary Table S1). The analysis reveals a series of core high-impact journals with groundbreaking significance in this field. Spine is indisputably defined as the core of the network and the basic knowledge library of the field with 25,786 unparalleled co-citations. As a supplement to this core, other top spine journals have also played an important role, such as the highly productive European Spine Journal (239 articles, 9,428 co-citations) and the highly influential The Spine Journal (impact factor IF 4.9). Meanwhile, the network itself also highlights the key specialized subdivisions. In terms of the publication of new research results, World Neurosurgery occupies a dominant position, ranking first with 296 articles. It is closely followed in academic influence by Journal of Neurosurgery: Spine, both of which together constitute a dual-hub for publishing the latest discoveries in the field. In addition, the outstanding performance of Pain Physician (175 articles published, 6,236 co-citations) further confirms the key role played by interventional pain management in LDH research.

**Figure 4:**
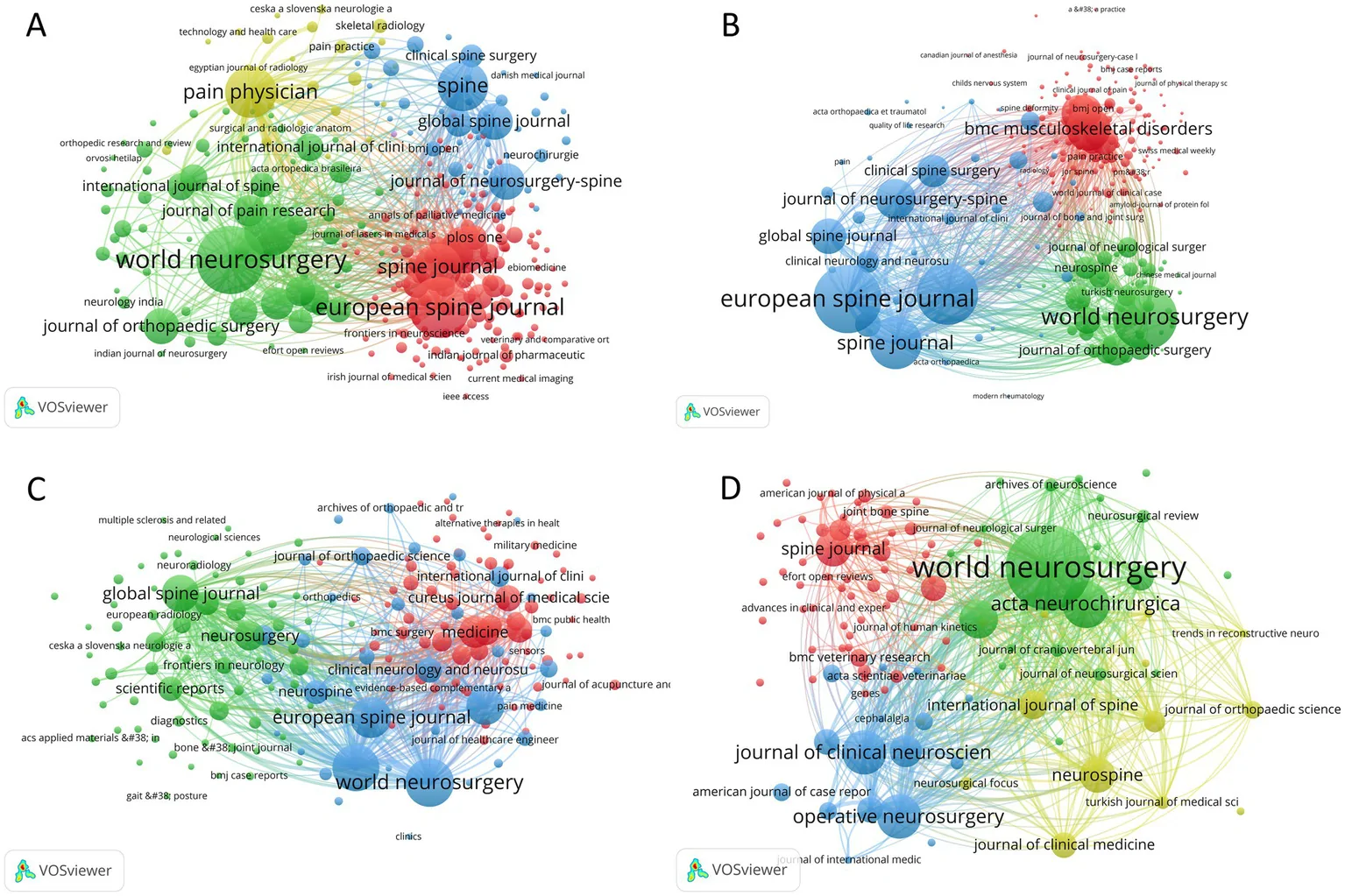
**(A)** Journal coupling network for lumbar disc herniation; **(B)** Journal coupling network for lumbar spinal stenosis; **(C)** Journal coupling network for cervical spondylosis; **(D)** Journal coupling network for thoracic disc herniation.

As determined by the journal coupling analysis (Figure 4B; Supplementary Table S1), the intellectual base of lumbar spine disease is mainly defined by a group of relatively fixed core journals. These key journals include European Spine Journal, Spine, The Spine Journal, and Journal of Neurosurgery: Spine. Although these journals have always performed prominently in many indicators (such as publication volume, citation influence, and network centrality), their specific contributions vary between research fields, and there are significant differences between the fields of lumbar disc herniation (LDH) and lumbar spinal stenosis (LSS). Although the European Spine Journal (329 articles) and Spine (301 articles) are the most prolific journals in LSS studies, the publication dominance in the LDH field has shifted significantly. In the latter field, World Neurosurgery (296 articles) took the lead, followed by the European Spine Journal (239 articles). However, citation analysis shows a more stable hierarchical structure of academic influence. Its top co-citation frequency (27,924 and 25,786 in the fields of LSS and LDH respectively) confirms that the journal Spine has become an unshakable academic cornerstone in these two fields. In addition to the core influence of Spine, two other journals have also shown significant cross-field influence: European Spine Journal (co-cited 10,346 times in the LSS field; 9,428 times in the LDH field) and The Spine Journal (a total of 7,325 times in the LSS field; 5,845 times in the LDH field). In addition, the academic authority of these journals is highly related to their performance indicators, which is intuitively reflected in their higher impact factors and their JCR quartile (Q1/Q2) ranking. In general, these data show that the whole picture of journals in the field of lumbar spine research shows the characteristics of both maturity and high concentration. The analysis reveals a key feature: although the publication volume is distributed in a group of core journals, its citation influence is highly concentrated in a smaller core subset, and Spine is at the top of the influence pyramid. This convergence of influence further confirms its foundational position in the process of building the knowledge base of LDH and LSS.

As visualized by the journal coupling network (Figure 4C), in the field of (CS), the importance of journals such as World Neurosurgery, European Spine Journal (blue cluster core), Global Spine Journal (green cluster core), and Neurosurgery and The Spine Journal is fully demonstrated by their huge node sizes, close connections, and core positions in their respective clusters. The data in Supplementary Table S1 further confirms this point. In terms of the publication volume, World Neurosurgery topped the list with 302 articles, followed by Spine (284 articles) and European Spine Journal (208 articles); in terms of the citation influence, Spine (24,294 co-citations in total) is in an absolute leading position, and its excellent performance highlights its foundational role. It is followed by other high-impact journals in the same echelon, namely European Spine Journal (8,565 co-citations), Journal of Neurosurgery: Spine (6,642 co-citations) and The Spine Journal (5,633 co-citations in total). The core network of knowledge production and dissemination in (CS) research is composed of these excellent journals. Their comprehensive performance in many indicators such as extremely high publication volume, extensive influence, strong impact factors and top JCR quartile ranking (mainly Q1/Q2 quartiles) further confirms their top academic status.

Unlike the research panorama of more common spinal diseases, the journal analysis of thoracic disc herniation (TDH; Figure 4D; Supplementary Table S1) depicts a research field characterized by low publication volume and a high concentration of neurosurgical literature. The network is built around several key hubs, and the visual analysis shows that they are World Neurosurgery, Acta Neurochirurgica and Journal of Clinical Neuroscience, respectively. The data in Supplementary Table S1 quantitatively confirms the dominant position of professional journals in disseminating the latest research results on TDH. Specifically, Journal of Clinical Neuroscience maintains the lead in the publication volume (89 articles), while World Neurosurgery relies on its considerable output and direct citation influence (276 citations) to stand out. On the other hand, the network also reveals another dimension of influence. Although The Spine Journal has a low volume of direct articles in this field, it occupies a key node in the network and shows a very high overall co-citation frequency (2,386 co-citations in total). This shows that its key role is not to serve as the main publication venue for TDH papers, but as a foundational source of references for the whole field. In summary, TDH’s journal panorama can be described as a “mixed ecosystem.” New case reports and technical notes in this field are mainly published in specialized neurosurgical journals such as Journal of Clinical Neuroscience and Acta Neurochirurgica. At the same time, the field relies on top journals with wider coverage and higher influence, such as The Spine Journal, to provide a theoretical and methodological foundation—a hybrid structure that is a typical feature of rare and highly technical disease research (Figure 5).

**Figure 5:**
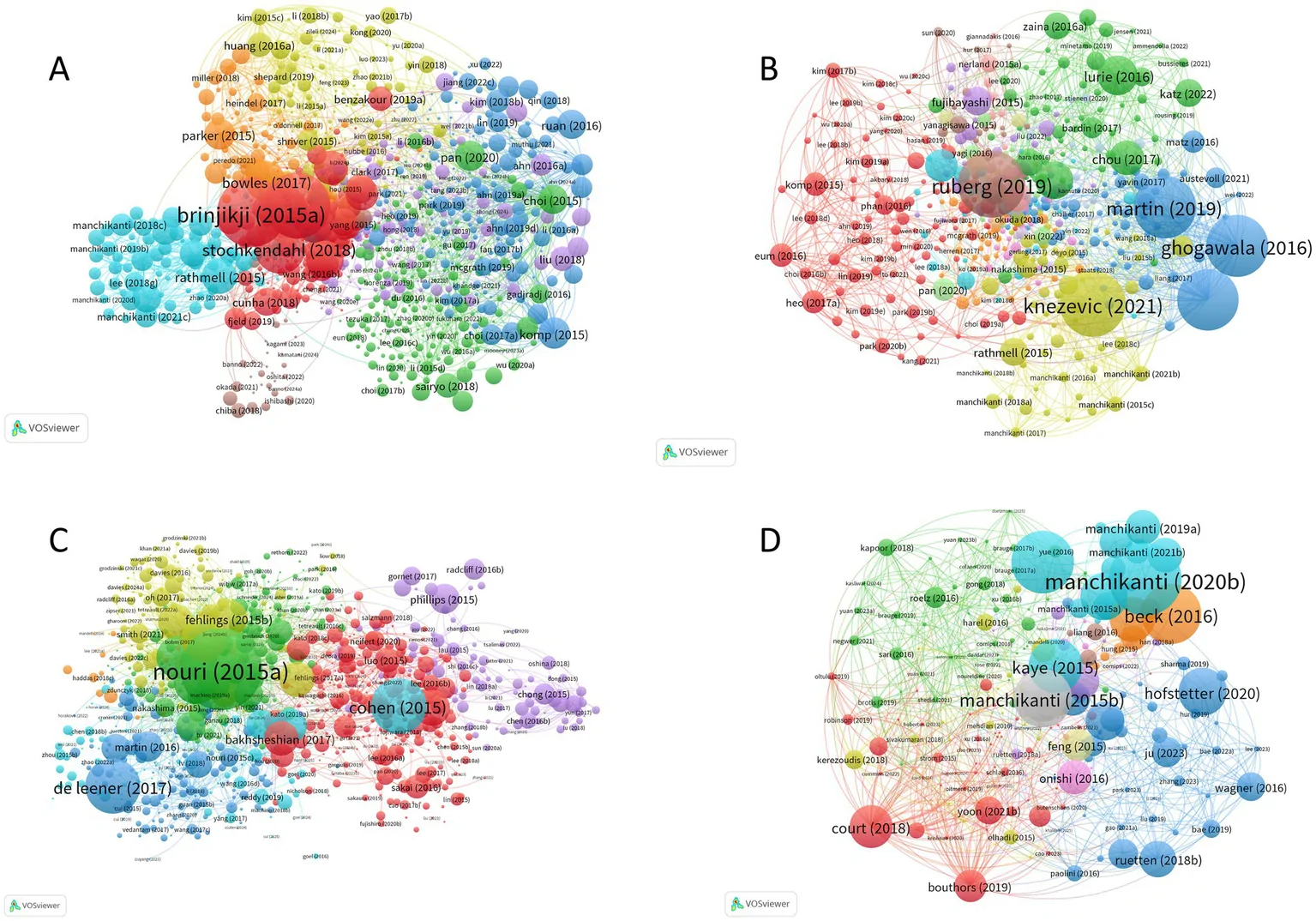
**(A)** Publication citation network analysis for lumbar disc herniation; **(B)** Publication citation network analysis for lumbar spinal stenosis; **(C)** Publication citation network analysis for cervical spondylosis; **(D)** Publication citation network analysis for thoracic disc herniation.

Through document co-citation analysis (Figure 6), we have identified the independent and interrelated knowledge pillars that constitute the knowledge base of these four major spinal diseases. Pioneering works in the field of standardized diagnosis, large-scale evidence-based clinical trials comparing different treatment modalities, cutting-edge breakthroughs in surgical technology, and the standardization of interventional pain management together constitute a solid foundation for this knowledge base. All of these have created an amazing depth and breadth in the field of lumbar disc herniation (LDH) research. This vast knowledge base is built around three core pillars: (1) Diagnostics: represented by the pioneering study of the standardized MRI grading system proposed by Pfirrmann in 2001. (2) Evidence-based medicine: established by key clinical trials such as the SPORT study (11) and the Dutch trial (12). (3) Surgical innovation: marked by the pioneering research of Ruetten (13) and Yeung and Tsou (14) in the field of endoscopy. The knowledge base of lumbar spinal stenosis (LSS) shares a common base with LDH, which is also firmly based on the principles of evidence-based medicine and advanced diagnostic methods. The low prevalence of thoracic disc herniation (TDH) determines that its evidence base must have a different structure. A key difference is that it lacks a foundation based on large-scale clinical trials, which is a typical feature of the more common spinal pathologies. Due to the lack of large-scale trials, the knowledge base of TDH has evolved into the product of the intersection of three key research fields: influential surgical reports (such as (15, 16)), improved minimally invasive techniques (such as (13)), and standardized interventional pain management protocols (such as (17)).

**Figure 6:**
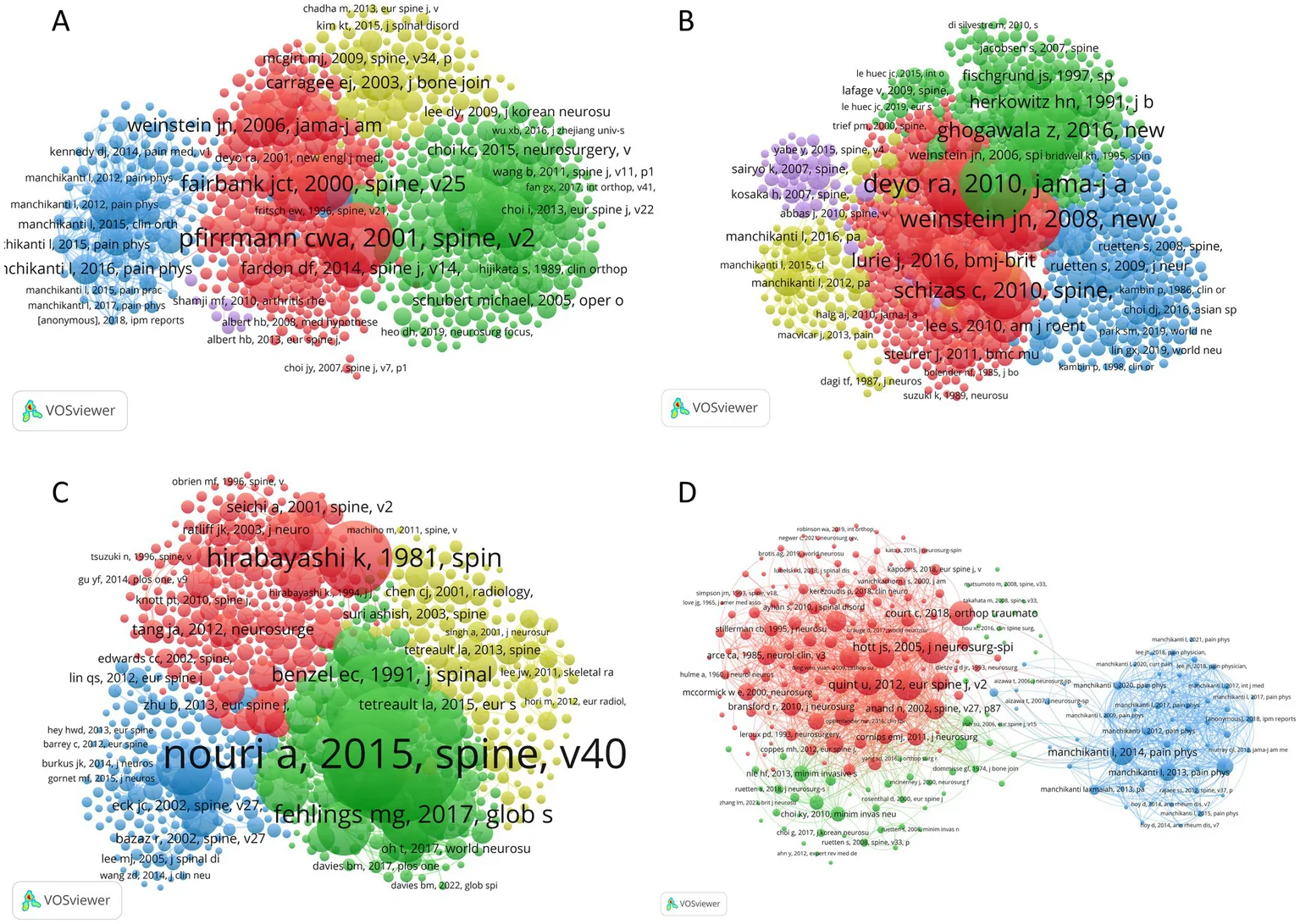
**(A)** Document co-citation analysis network for lumbar disc herniation; **(B)** Document co-citation analysis network for lumbar spinal stenosis; **(C)** Document co-citation analysis network for cervical spondylosis; **(D)** Document co-citation analysis network for thoracic disc herniation.

The structure of the thoracic disc herniation (TDH) keyword co-occurrence network (Figure 7A) is defined by a problem-oriented core axis from diagnosis to surgical management. The core axis is formed by the close integration of two major research clusters. One is the red cluster, which represents the clinical problem itself. Its core terms include “thoracic disc herniation”, “surgery” and its main clinical consequence “thoracic myelopathy.” The second is the green cluster, which outlines the diagnostic process and is characterized by general diagnostic terms such as “disc herniation,” “magnetic resonance imaging” and “thoracic.” The close connection between these two clusters is manifested in the evolution trajectory from “magnetic resonance imaging” to “thoracic disc herniation” and finally to “myelopathy,” which deeply reveals the core research problems in this field. In short, this field mainly focuses on the use of imaging methods to identify herniations leading to neurological deficits, and then discusses surgery as the main therapeutic intervention.

**Figure 7:**
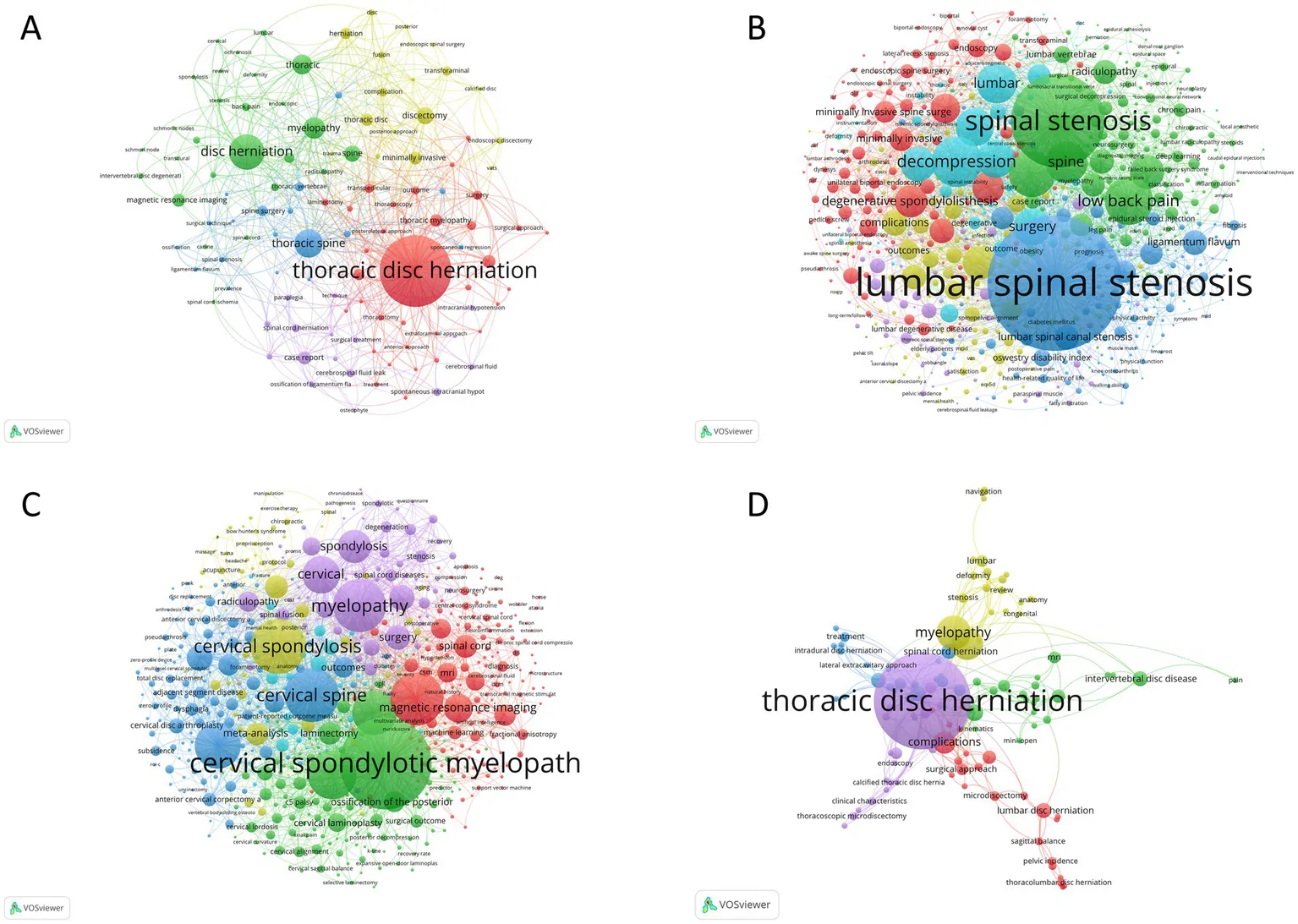
**(A)** Keyword co-occurrence network analysis for lumbar disc herniation; **(B)** Keyword co-occurrence network analysis for lumbar spinal stenosis; **(C)** Keyword co-occurrence network analysis for cervical spondylosis; **(D)** Keyword co-occurrence network analysis for thoracic disc herniation. In these networks, each node represents a keyword, with its size proportional to its occurrence frequency. The lines indicate co-occurrence relationships, where thicker lines represent stronger connections. Varying colors denote distinct thematic clusters identified by the algorithm.

Although the thoracic disc herniation (TDH) network (Figure 7A) has segment specificity, the lumbar spinal stenosis (LSS) network (Figure 7B) represents a more multidimensional and more comprehensive research paradigm. The core of the network is dominated by three tightly intertwined large clusters. The blue cluster focuses on the disease entity of “lumbar spinal stenosis” itself and its functional impact (evaluated by tools such as “Oswestry Disability Index”). The network has drawn clear boundaries, and the green cluster focuses on clinical manifestations such as “low back pain” and “nerve root disease”, as well as non-surgical treatment plans such as “epidural steroid injection”. Finally, the research topic of the red/cyan cluster covers surgical strategies (“decompression”), advanced technology (“minimally invasive spinal surgery”) and related pathological conditions (“degenerative spondylolisthesis”). The extremely dense and stable connections between these three core nodes and their clusters constitute the skeleton of the whole network. This shows that the organization of LSS research promotes a comprehensive understanding and transcends isolated views on diseases, symptoms and their treatments. This comprehensive research method marks a mature, patient-centered field that is committed to optimizing the clinical outcomes of this complex multifaceted pathological state.

Contrary to the multidimensional and distributed structure of the LSS keyword network (Figure 7B), the network of CS (Figure 7C) is highly centralized. The highly centralized topology of the network is based on the dominant core of pathological terms (such as “cervical spondylosis”) and is surrounded by satellite clusters representing diagnosis, intervention and symptoms, which strongly implies a specific research paradigm. Under this paradigm, neurological impairment is conceptualized as a direct sequela of the age-related disease process. Therefore, the research work mainly shows the characteristics of dual focus: first, it is committed to accurate diagnosis, mainly relying on imaging methods such as MRI; second, we are committed to the application of minimally invasive neurodecompression to relieve neural structure compression and optimize the neurological outcomes of elderly patients.

The keyword co-occurrence network of thoracic disc herniation (TDH) is characterized by a unique dual-core topology. One of the cores represents the disease entity itself (“thoracic disc herniation”), while the other core marks its main clinical consequences (“myelopathy”). The prominent position of the closely related keyword “spinal cord hernia” further strengthens its potential pathological mechanism as a direct spinal cord compression. Around the core pathological area, there is a major cluster related to diagnosis. The MRI node is located at the center of the cluster and acts as a key hub for connecting core pathology with broader concepts, including general diagnostic terms such as “disc disease” and patient reports such as “pain.” The clustering of keywords related to intensive treatment is also very significant. It not only contains conventional terms such as “surgical methods,” “treatment” and “complications,” but also covers specific minimally invasive techniques such as “endoscopy” and “thoracoscopic minimally invasive discectomy”. In addition to the clinical core in the network, the emerging research frontier is also clearly visible. The common appearance of terms such as “lumbar disc herniation” shows the comparative research perspective of different spinal segments. In addition, the emergence of keywords such as “sagittal balance” and “pelvic incidence” reflects the growing interest of the academic community in spinal biomechanics. In short, the TDH research network shows that although the field is deeply rooted in clinical orientation (diagnostic and surgical management), it has begun to expand into the exploration of comparative anatomy and biomechanical factors. The key word emergence analysis of lumbar spinal stenosis (LSS) research (Figure 8B) clearly shows the main evolution trajectory from basic concepts to high-tech intervention.

**Figure 8:**
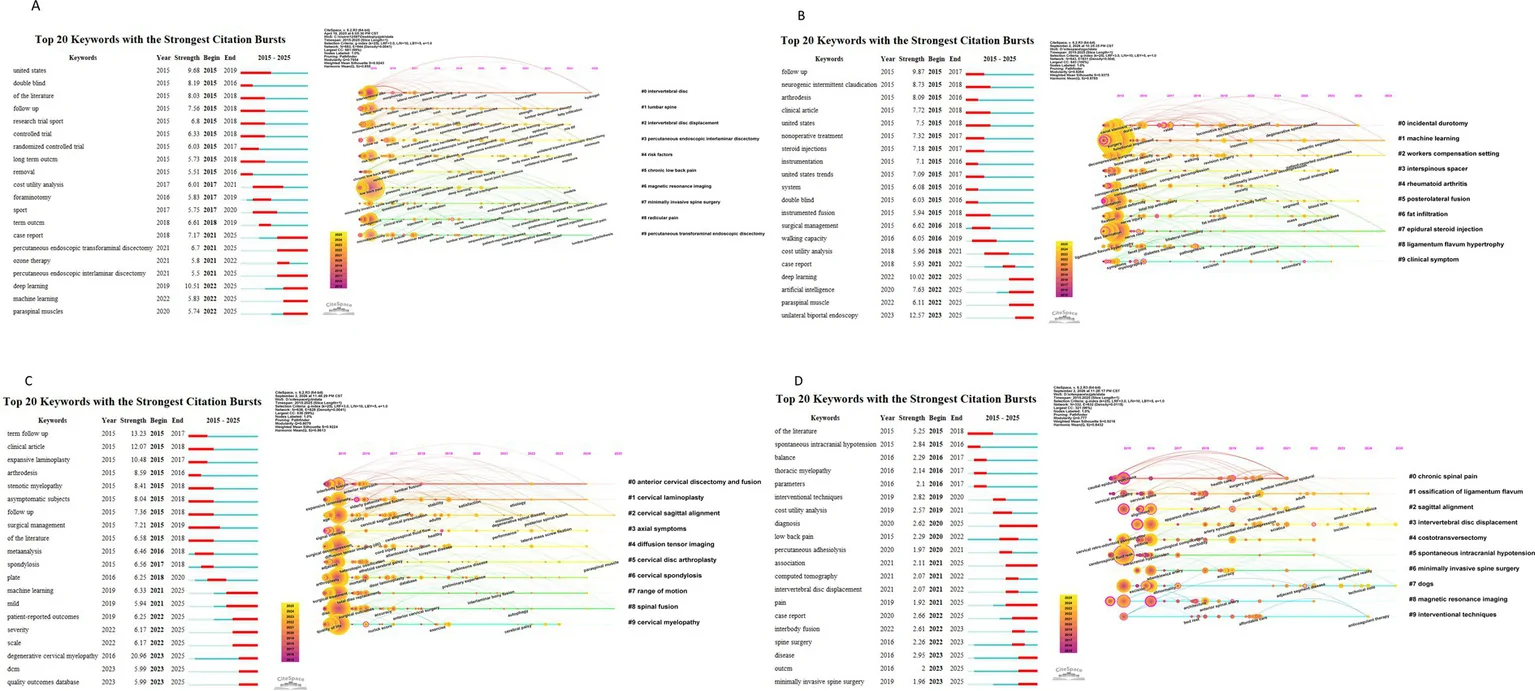
Temporal trends in spinal degenerative disease research: a comparative analysis of the top 20 most cited keywords in lumbar disc herniation, lumbar spinal stenosis, cervical spondylosis and thoracic disc herniation. **(A)** Top 20 most cited keywords in lumbar disc herniation research; **(B)** Top 20 most cited keywords in lumbar spinal stenosis research; **(C)** Top 20 most cited keywords in cervical spondylosis research; **(D)** Top 20 most cited keywords in thoracic disc herniation research. In the left panels, “Strength” reflects the citation burst intensity. On the timeline, the blue line represents the overall study period, while the red line segment highlights the specific burst duration. The right panels display the corresponding CiteSpace networks. Each node’s overall size reflects its total occurrence frequency. The nodes display a “tree-ring” structure, where the colors of the concentric rings represent the occurrence frequency across different years (cooler colors for earlier years, warmer colors for recent years), visualizing the temporal evolution of research hotspots.

The early stage (about 2015–2018) focused on establishing core clinical characteristics and formulating research standards. The classic symptom “neurogenic intermittent claudication” (burst strength 8.73) and the strong emergence of the traditional therapy “spinal fusion” (burst strength 8.09) reflect the research focus of this period. In the early stage, it also showed great importance to methodological rigor, which was reflected in the high-intensity emergence of keywords such as “follow-up” (9.87) and “double blind” (6.03). The mid-term stage (about 2016–2021) represents a key transition period for the development of this field, and the research focus shifts from the definition of the disease to the evaluation of functional outcomes after intervention. The emergence of keywords such as “surgical management” (surgence intensity 6.62) and key patient-reported outcome “walking ability” (surgency intensity 6.05) shows that research is paying more and more attention to patient-centered outcome indicators. The current research trajectory (from about 2020 to the present) is characterized by the strong momentum of technological innovation (especially in the field of minimally invasive surgery). The strong emergence of unilateral biportal endoscopy (UBE) (burst strength 12.57) marks the peak of this trend and constitutes the hottest research frontier at present. The development path from clinical foundation to advanced surgical solutions fully confirms the continuous maturity of the LSS research field.

As shown in Figure 8C, the keyword burst analysis of CS outlines a clear evolutionary trajectory. This field has changed from focusing on basic surgical techniques to a more refined, patient-centered and data-driven research paradigm. The emergence of important keywords confirms that the early stage of the study (about 2015–2018) mainly focused on the core surgical challenges. These prominent words include intervention in specific surgical procedures (burst intensity 10.48) and its target indication “spinal cord disease” (burst intensity 8.41). In the mid-term stage (about 2018–2021), the focus of the research shifted to the specific details of surgical hardware and implants, and the appearance of the keyword “plate” (burst intensity 6.25) confirmed this trend. In the recent research stage (about 2021 to the present), “degenerative cervical myelopathy” (DCM) has become a unified core concept, integrating the previous independent research direction. Nowadays, contemporary DCM research is characterized by the integration of multi-dimensional methods, including the results of patient reports (burst intensity 6.25), objective indicators of disease severity (burst intensity 6.17) and advanced analysis technologies such as machine learning. This evolutionary trajectory clearly shows that driven by the ability of large-scale data analysis, the paradigm of this field has changed to neuroprotection for aging.

The key word emergence analysis of thoracic disc herniation (TDH; Figure 8D) clearly outlines the mature process of this emerging research field from macro knowledge integration to a more organized clinical focus. In the early stage (about 2015–2018), the research focused on establishing basic knowledge. The uniqueness of this basic stage lies in the general keyword “literature” (literature, burst intensity 5.25, marking a broad review stage) and the core clinical problem “thoracic myelopathy” (thoracic myelopathy, burst intensity 2.14) appearing at the same time. The mid-term stage (about 2019–2021) represents the pragmatic transition period of the development of this field, and clinical application and evaluation have received more and more attention. During this period, the focus of research continued to expand, not only covering specific “intervention techniques” (burst intensity 2.82), but also involving two other key areas: economic assessment through “cost-utility analysis” (burst intensity 2.57), and progress in imaging diagnostics such as computed tomography (CT).

The research characteristics of the recent stage (about 2020 to the present) are developing towards higher accuracy and depth of these two dimensions. This evolution path from literature integration to structured focus on evidence-based patient care fully reflects the gradual maturity of the field of TDH research.

## 4 Discussion

### 4.1 The panoramic and dynamic evolution of the study of the four major spinal degenerative diseases revealed by bibliometrics

As far as we know, this study is the first to conduct a simultaneous, comparative bibliometric analysis to macroscopically and quantitatively portray the research patterns and evolutionary trajectories of the four key spinal degenerative diseases (LDH, CS, LSS, and TDH) within a unified framework. The shared knowledge structure constitutes the core of these research fields. Keyword analysis fully confirms this point, which reveals that each disease has a common basic pillar in terms of diagnostics (“MRI”), clinical manifestations (“myelopathy,” “radiculopathy”) and therapeutic intervention (“surgery” and “minimally invasive spinal surgery”). The time series analysis of keyword bursts clearly outlines the dynamic evolution of this field, which is also one of the key aspects of this study. This evolutionary path marks a decisive shift, that is, the research focus is gradually abandoning basic methodologies and traditional treatment methods to embrace the new research paradigm. A landmark trend in modern age-related spinal degenerative disease research is reflected in the intersection and integration of three core pillars: minimally invasive neuro-decompression technology, intelligent precision technology, and high attention to the neurological functional outcomes reported by patients. These bibliometric trends directly reflect actual treatment priorities: addressing the unmet clinical need to minimize surgical morbidity in the frail aging population, thereby guiding surgeons in everyday clinical decision-making.

### 4.2 Insight into future research directions based on knowledge atlas: deepening understanding and technological innovation

The bibliometric and knowledge atlas analysis presented by this study have made dual contributions. Our research results not only provide a macro-panorama of the four major spinal degenerative diseases, but also depict their complex knowledge structures, research hotspots and evolutionary trends (Figures 7, 8). The ultimate value of this detailed atlas is that it provides a data-driven research roadmap for the academic community.

#### 4.2.1 In-depth exploration of pathological mechanisms and prognostic subtyping

Based on the specific knowledge gaps and emerging trends for each disease, we put forward the following targeted research agenda. The study of lumbar disc herniation (LDH) needs to achieve a paradigm shift, so as to go beyond the traditional mechanical perspective of focusing only on disc herniation. The continuous burst of the keyword “sagittal balance” (Figure 8A) points out the key direction of future research: an in-depth exploration of how biomechanical factors affect the development and prognosis of LDH. In order to better understand the pathophysiological mechanism of intervertebral disc degeneration and find new repair targets, it is crucial to incorporate molecular biological methods into future research. Represented by recent research findings (Figure 8A), the evidence regarding the paravertebral muscle is increasing, which highlights the need to clarify its exact functional role in maintaining spinal stability and promoting patient recovery. A key challenge in the study of lumbar LSS is to link specific pathological drivers with clinical results. Establishing this association is crucial for formulating individualized treatment strategies. It is urgent to clarify the potential molecular mechanisms of pathological processes such as ligamentum flavum hypertrophy, because these processes promote the occurrence of common comorbidities, as shown in “degenerative spondylolisthesis” (Figure 7B). To lay a solid foundation for personalized treatment strategies, it is necessary to integrate specific clinical manifestation data such as this pathological cognition and anatomical subtype (such as central canal stenosis and lateral recess stenosis) and typical “neurogenic intermittent claudication” (Figure 8B). The current (CS) research is highly concerned about “degenerative cervical myelopathy” (DCM; Figure 8C), which creates opportunities and needs for the establishment of a forward-looking research agenda. Priority should be given to finding biomarkers for early diagnosis and clarifying the mechanism of nerve damage and repair, especially the neuroinflammatory microenvironment and the regenerative potential of the aging central nervous system. For patients at different stages of “severity” (Figure 8C), determining the natural course of their disease and the best time for intervention depend heavily on these cognitions. The exploration of special pathologies, especially OPLL (posterior longitudinal ligament ossification), must continue to be the focus. This is not only because of its clinical significance, but also due to the important research contributions made in this field in history (Figure 3C). Since thoracic disc herniation (TDH) is relatively rare and the research volume is small (Figure 1), establishing basic cognition is the top priority in this field. In order to promote the understanding and management of TDH, future research must focus on several key goals: clarifying its natural course of disease, clarifying the mechanism of occurrence of special types (such as calcified protrusion, “spinal cord herniation”; Figure 7A), and identifying high-risk factors, so as to improve early “diagnosis” and risk stratification (Figure 8D).

#### 4.2.2 Comprehensive research on disease interaction, overall biomechanics and systemic impacts

One of the main limitations of our independent analysis of each disease is that it fails to fully reflect the common clinical reality of multi-segmental degeneration and disease coexistence. Future research needs to shift to a more holistic spinal perspective. In-depth exploration of the correlation of multisegmental degradation is the top priority of future research. When degenerative diseases coexist in different segments (such as “lumbar disc herniation”; Figure 7D), it is urgent to systematically explore their epidemiological characteristics, interaction mechanisms and cumulative effects on spinal function and quality of life. Assessing the impact of overall spinal balance is another key area for future research. It is crucial to understand the role of the sagittal plane (for example, “sagittal balance”; Figure 7D) and the imbalance of the coronal plane, because they will affect the pathogenesis of the disease, guide treatment strategies and determine the long-term efficacy. Future research must also pay due attention to systemic factors. Future research must go beyond local pathology to deal with systemic factors, which requires the joint efforts of the academic community to identify relevant contributing diseases (such as osteoporosis), assess their impact on the degeneration process and the risk of complications, and formulate corresponding comprehensive management strategies.

#### 4.2.3 Innovation of intervention strategies: towards precision, minimally invasive, intelligence and comprehensive rehabilitation

A key finding of our analysis is that a new paradigm of more comprehensive, precise and patient-centered intervention is emerging. The evolution of these strategies may revolve around four major frontier areas: surgery and technological innovation, comprehensive perioperative care, evidence-based non-surgical programs, and comprehensive rehabilitation. The burst of “minimally invasive spine surgery” (Figure 8D), especially advanced endoscopic technologies such as PETD and UBE (Figures 8A,B), especially advanced endoscopic technologies such as PETD and UBE (Figures 8A,B) foreshadow an obvious trend aimed at achieving full neural decompression while minimizing iatrogenic physical trauma and neurological complications in the elderly. For practicing surgeons, these publication metrics provide direct evidence to justify selecting endoscopic techniques over traditional open surgeries for vulnerable patients. The integration of artificial intelligence (AI) is expected to further expand this trend. However, it is vital to differentiate that while AI exhibits massive keyword bursts indicating a strong research hotspot, its clinical evidence requires further qualitative evaluation. The AI-assisted decision-making support system driven by “deep learning” and “machine learning” (Figures 8A,C) will inevitably contribute to the realization of highly individualized treatment plans. These systems will assist in selecting the best surgical approach by analyzing the patient’s unique pathology, symptoms and imaging data. The systematic promotion of ERAS (Enhanced Recovery After Surgery) in spinal surgery responds to the growing concern about patient experience in this field, and the burst of “patient-reported outcomes” (Figure 8C) further highlights this. The future of non-surgical treatment that goes hand in hand with surgery must be based on rigorous evidence-based practice. Therefore, it is urgent to carry out high-quality research to clearly define the real efficacy, long-term value and precise indications of existing physical therapy, drug regimens and interventional operations such as “steroid injection” (Figure 8B). This attention to evidence-based practice will pave the way for the innovation and verification of new conservative treatment strategies. Finally, without the deep integration of rehabilitation science, a truly comprehensive treatment model cannot be achieved. The recent burst of the keyword “paraspinal muscles” (Figures 8A,B) marks that people are increasingly aware of its key role in biomechanical stability.

#### 4.2.4 Expansion of research paradigms and acceleration of translational outcomes

Based on the results of this bibliometric analysis, this paper proposes a multi-dimensional strategy aimed at promoting the research of spinal diseases. The primary goal of future research is to improve the scale and generalizability of clinical evidence. As shown in Table 1, the geographical concentration presented by the study is a limitation that must be addressed. Strengthening international cooperation and multi-center research is crucial to achieving this goal. Carrying out large-scale real-world studies (RWS) required to assess long-term outcomes is extremely dependent on such cooperation. The recent burst of the “Quality Outcomes Database” (Figure 8C) confirms the development trend of such research. While striving to broaden clinical evidence, we must move towards the second frontier of therapeutic innovation, especially in the field of biomaterials and regenerative medicine. The high attention to “disc arthroplasty” (Figure 7C) proves the open attitude of this field to new technologies. The crucial next step is to explore advanced bone grafting materials, tissue engineering and regenerative therapy in depth within the framework of enhanced translational medicine and implementation science. The ultimate goal is to ensure the effective translation of innovative results (covering both methodology and technology) from scientific research to clinical practice. In order to bridge the long-standing “know-do gap” and ensure that research breakthroughs yield tangible patient benefits, it is imperative to systematically overcome the real-world barriers to clinical implementation. Primarily, advanced endoscopic techniques exhibit a notoriously steep learning curve, necessitating rigorous, structured training paradigms. Furthermore, the widespread adoption of spinal robotics and AI-driven systems is significantly bottlenecked by exorbitant initial costs, heterogeneous insurance reimbursement policies, and stringent regulatory hurdles surrounding the “black-box” nature of medical AI. Crucially, translating these innovations to the geriatric population poses unique challenges. Elderly patients frequently present with severe osteoporosis and complex anatomical deformities, mandating that novel AI algorithms, robotic trajectories, and biomedical implants be specifically calibrated for fragile spinal structures. Addressing these multi-dimensional translational barriers is the absolute prerequisite for fulfilling the clinical utility of our proposed roadmap.

### 4.3 Implications of data-driven diagnosis and treatment models for the optimization of clinical practice

By revealing key research trends (Figure 8) and core concerns (Figure 7), this bibliometric analysis provides a macroscopic, evidence-based roadmap to guide clinical practice towards a more comprehensive, accurate and patient-centered management model.

#### 4.3.1 Upgrading clinical evaluation systems: from static imaging to multi-dimensional dynamic early warning

Mature diagnostic techniques (such as MRI; Figure 7) intersect with increasing attention to functional outcomes (Figures 8B,C), which inevitably requires future clinical evaluations to go beyond static imaging and shift to multi-dimensional risk stratification and active screening. Therefore, it is imperative to evolve the strategy of patient evaluation and monitoring. The characteristic feature of this new method is that it relies on a comprehensive evaluation of comprehensive multi-source data. This comprehensive assessment includes the combined application of detailed medical history collection, neurological examination and standardized functional scales (such as the widely cited Oswestry Disability Index; Figure 7B; Supplementary Table S2), with special attention to tracking the trajectory of neurological decline in the elderly. With the help of advanced diagnostic techniques, the clinical situation of patients should be further enriched in order to provide a more comprehensive perspective for disease assessment. These advanced diagnostic techniques include dynamic imaging, special magnetic resonance sequences, and the evaluation of key biomechanical parameters such as sagittal plane balance. While carrying out the above-mentioned comprehensive assessment, we must implement active management strategies for pre-symptomatic patients and confirmed patients. Targeted early screening should be carried out in high-risk groups (individuals with family or occupational risk factors) in order to determine the indications for early clinical intervention for the disease in a timely manner. Regular dynamic monitoring is crucial for patients who have been diagnosed, especially those who are receiving conservative treatment. This continuous assessment is critical to achieving two key objectives: timely detection of the progression of the disease and timely adjustment of the evidence-based basis for treatment plans.

#### 4.3.2 Formulation of individualized treatment decisions and optimization of multimodal treatment tools

Future treatment choices must be guided by a sophisticated, individualized philosophy. Although our analysis confirms that surgery continues to occupy a central position (Figure 7), this (shift toward individualization) is still a key conclusion. Bridging these findings with clinical practice, this shift dictates that a surgeon’s decision-making process must move beyond simple anatomical decompression to focus on actual functional recovery. This new method is supported by three core principles: the strategic application of minimally invasive and effective interventions, the standardization of evidence-based conservative treatment, and the full use of multidisciplinary expertise in complex cases. As the basic principle of the stepped care model, it is advocated to give priority to the least invasive but effective treatment plan. At the same time, standardized and evidence-based treatment methods must be adopted for patients who are suitable for non-surgical management. For extremely complex cases, multidisciplinary team discussion (MDT) is crucial for formulating the best strategy. By integrating diversified expertise from different professions, this collaborative model ensures the development and implementation of the most reliable and patient-centered treatment plan.

#### 4.3.3 Construction of an integrated diagnosis and treatment model: MDT promotion and comprehensive rehabilitation

As intuitively presented in the complex network diagram (Figure 7), the high complexity of spinal diseases inevitably requires a fundamental shift from an isolated specialist model to a truly integrated medical model. This patient-centered paradigm is defined by three expanding dimensions: the specialist team, the entire patient journey, and the broader medical and health system. In order to provide patients with seamless one-stop evaluation and collaborative management and break down traditional specialist barriers, standardized multidisciplinary teams (MDTs) form the core of this model. The team must bring together experts from the fields of spinal surgery, pain medicine, rehabilitation medicine, imaging and neurology. This model requires the deep integration of rehabilitation services into all clinical stages, including prehabilitation and subsequent long-term functional support. This continuous method plays a crucial role in greatly improving the long-term quality of life of patients. Finally, this integration is further extended, closely connecting specialist centers with a broader primary healthcare network.

### 4.4 Advantages, limitations and core innovation

This study has built a macro framework aimed at depicting the panorama of the study of the four major spinal degenerative diseases at the same time, and on this basis, a novel cross-pathological comparative analysis was carried out. This constitutes a prominent feature of the current research and enhances its overall contribution. By visualizing knowledge into a map, researchers can go beyond the limitations of simple publication counts, so as to gain an in-depth insight into the knowledge structure and development trajectory of a certain field. However, it must be recognized that the method of bibliometrics is not without its inherent limitations. This analysis is based on the data of Web of Science, Scopus and PubMed databases, so its coverage is mainly limited to English literature. This English-language and database restriction constitutes a recognized methodological limitation. As our results show that China is a major producer of research in this field, the exclusion of significant non-English studies existing in local databases (such as CNKI) inevitably introduces a degree of language bias. Consequently, this exclusion might partially skew the interpretation of the true ‘global’ landscape. While our analysis successfully captures the international mainstream trajectory, it may underrepresent valuable region-specific clinical realities, localized technological innovations, and domestic consensus guidelines published in other languages. Furthermore, It must be emphasized that this analysis provides a descriptive overview of the research activities, not a direct verification or qualitative evaluation of clinical evidence, we map the “research hotspots” (what is currently being studied) rather than the “clinical evidence” (what has been proven effective). The value of our research results lies in their ability to provide strategic guidance in the field of research, thus complementing rather than replacing evidence integration methods such as systematic reviews and meta-analysis.

This study redefines the function of bibliometrics in spinal medicine, transforming it from a retrospective evaluation tool to a forward-looking tool to guide future research. This is achieved through three independent but closely connected contributions. (1) This study establishes a comprehensive cross-pathological framework, integrating the knowledge between previously isolated disciplines. There is a significant imbalance in the research progress in different fields of spinal pathology. Our research confirms this by shifting from the traditional single disease focus to a parallel analysis of four diseases. For example, the analysis highlights the comparison between technology-driven progress (i.e., endoscopic technology) in lumbar spine diseases and concept-driven conceptual integration in cervical myelopathy. Beyond simple comparisons, this analytical framework provides a theoretical basis for a new research paradigm: that is, the strategic transfer of successful innovative technologies and conceptual models between different anatomical parts. (2) The novel methodology proposed in this article marks a key shift in strategic forward-looking by replacing subjective expert forecasts with objective and quantitative predictions. Our method converts bibliometric signals into actionable outputs—that is, a series of carefully sorted high-impact research problems and clinical optimization strategies, which have been discussed in detail in Sections 4.2 and 4.3. This method establishes a new epistemological framework for identifying future research directions and determining its priorities, thus triggering a paradigm shift in this field. (3) A key contribution of this study is to build a comprehensive evidence-based framework to provide guidance for future priorities of spinal care. Through a comprehensive analysis of various research trends, we can clearly see that the research focus in this field is undergoing fundamental changes. The new paradigm takes the “trinity” of interventions (minimally invasive, intelligent and biomechanical guidance) as the core; in this paradigm, success increasingly depends on patient-centered evaluation indicators. Our data-driven analysis enables the evolution trajectory of the specialty to be clearly defined based on empirical certainty, thus transcending the category of subjective speculation. Looking forward to the future, it is the responsibility of subsequent research to be consistent with the paradigm established in this article. Scientific research that fails to achieve this goal will face the risk of detaching from the continuous evolution and evidence-based development trajectory of the field. This work fundamentally redefines bibliometrics and pushes its application from retrospective analysis to forward-looking strategic forecasting.

## 5 Conclusion

This study conducts a systematic bibliometric analysis of four age-related key spinal degenerative diseases (lumbar disc herniation, lumbar spinal stenosis, cervical spondylosis and thoracic disc herniation). The scope of the study is limited to the literature published and indexed in three mainstream databases since 2015. Our analysis shows that the number of publications continues to grow, among which lumbar disease has become the most widely studied pathological type. China and the United States are particularly prominent, becoming dominant regional contributors and establishing their own unique international cooperation network.

Crucially, the core findings of this study show that the field is undergoing profound evolution and transformation. The research focus is shifting from traditional surgical and diagnostic methods to a comprehensive intervention model characterized by minimally invasive neural decompression, artificial intelligence (AI)-driven and biomechanical guidance methods tailored to the aging population. Endoscopic technology is a typical representative of this development trend. This evolutionary trend, coupled with the emerging attention to patient-centered neurological outcome indicators, inevitably requires the academic community to work together to use this momentum to lead the next wave of innovation. Future work must pay attention to the following aspects: (1) There is an urgent need to deepen the understanding of the underlying pathophysiological mechanism of nerve damage; (2) Strictly optimize and verify the specific minimally invasive treatment strategies for elderly patients; (3) Strengthen multidisciplinary cooperation and translational research.

The ultimate goal of these efforts is to harness recent technological and conceptual innovations to advance the prevention and treatment of common age-related spinal diseases, thereby improving geriatric neurological outcomes.

## Data Availability

The original contributions presented in the study are included in the article/Supplementary material, further inquiries can be directed to the corresponding authors.

## References

[1] Purushotham S Hodson N Greig C Gardner A Falla D. Microscopic changes in the multifidus muscle in people with low back pain associated with lumbar disc herniation. Sci Rep. (2024) 14:31927. doi: 10.1038/s41598-024-83373-9, 39738508 PMC11686315

[2] Iglesias P. An update on advances in hypopituitarism: etiology, diagnosis, and current management. J Clin Med. (2024) 13:16161. doi: 10.3390/jcm13206161, 39458112 PMC11508259

[3] Di X Chen J Li Y Wang M Wei J Li T . Crosstalk between fibroblasts and immunocytes in fibrosis: from molecular mechanisms to clinical trials. Clin Transl Med. (2024) 14:e1545. doi: 10.1002/ctm2.1545, 38264932 PMC10807359

[4] Furuse Y. Analysis of research intensity on infectious disease by disease burden reveals which infectious diseases are neglected by researchers. Proc Natl Acad Sci USA. (2019) 116:478–83. doi: 10.1073/pnas.1814484116, 30598444 PMC6329976

[5] Melendez-Torres GJ O'Mara-Eves A Thomas J Brunton G Caird J Petticrew M. Interpretive analysis of 85 systematic reviews suggests that narrative syntheses and meta-analyses are incommensurate in argumentation. Res Synth Methods. (2017) 8:109–18. doi: 10.1002/jrsm.1231, 27860329 PMC5347877

[6] Nason SL McCord J Feng YL Sobus JR Fisher CM Marfil-Vega R . Communicating with stakeholders to identify high-impact research directions for non-targeted analysis. Anal Chem. (2025) 97:2567–78. doi: 10.1021/acs.analchem.4c04801, 39883652 PMC11886761

[7] Weng R Lin DX Song YK Guo HW Zhang WS He XM . Bibliometric and visualized analysis of research relating to minimally invasive spine surgery reported over the period 2000-2022. Digit Health. (2023) 9:20552076231173562. doi: 10.1177/20552076231173562 PMC10164264 37163171

[8] Garner RM Hirsch JA Albuquerque FC Fargen KM. Bibliometric indices: defining academic productivity and citation rates of researchers, departments and journals. J Neurointerv Surg. (2018) 10:102–6. doi: 10.1136/neurintsurg-2017-013265, 28824008

[9] Magalhães T Dinis-Oliveira RJ Taveira-Gomes T. Digital health and big data analytics: implications of real-world evidence for clinicians and policymakers. Int J Environ Res Public Health. (2022) 19:8364. doi: 10.3390/ijerph19148364, 35886214 PMC9325235

[10] Voit EO. Perspective: dimensions of the scientific method. PLoS Comput Biol. (2019) 15:e1007279. doi: 10.1371/journal.pcbi.1007279, 31513575 PMC6742218

[11] Weinstein JN Lurie JD Tosteson TD Skinner JS Hanscom B Tosteson AN . Surgical vs nonoperative treatment for lumbar disk herniation: the spine patient outcomes research trial (SPORT) observational cohort. JAMA. (2006) 296:2451–9. doi: 10.1001/jama.296.20.2451, 17119141 PMC2562254

[12] Peul WC van Houwelingen HC van den Hout WB Brand R Eekhof JA Tans JT . Surgery versus prolonged conservative treatment for sciatica. N Engl J Med. (2007) 356:2245–56. doi: 10.1056/NEJMoa064039, 17538084

[13] Ruetten S Komp M Merk H Godolias G. Full-endoscopic interlaminar and transforaminal lumbar discectomy versus conventional microsurgical technique: a prospective, randomized, controlled study. Spine (Phila Pa 1976). (2008) 33:931–9. doi: 10.1097/BRS.0b013e31816c8af7, 18427312

[14] Yeung AT Tsou PM. Posterolateral endoscopic excision for lumbar disc herniation: surgical technique, outcome, and complications in 307 consecutive cases. Spine (Phila Pa 1976). (2002) 27:722–31. doi: 10.1097/00007632-200204010-00009, 11923665

[15] Hott JS Feiz-Erfan I Kenny K Dickman CA. Surgical management of giant herniated thoracic discs: analysis of 20 cases. J Neurosurg Spine. (2005) 3:191–7. doi: 10.3171/spi.2005.3.3.0191, 16235701

[16] Quint U Bordon G Preissl I Sanner C Rosenthal D. Thoracoscopic treatment for single level symptomatic thoracic disc herniation: a prospective followed cohort study in a group of 167 consecutive cases. Eur Spine J. (2012) 21:637–45. doi: 10.1007/s00586-011-2103-0, 22160099 PMC3326138

[17] Manchikanti L Abdi S Atluri S Benyamin RM. An update of comprehensive evidence-based guidelines for interventional techniques in chronic spinal pain. Part II: guidance and recommendations. Pain Physician. (2013) 16:S49–S283. doi: 10.36076/ppj.2013/16/S49 23615883

