# Supplementary material for: Age-related spinal degenerative diseases: a quantitative analysis of the dynamic evolution, knowledge structure, and research frontiers in neurological damage
Source: Front Neurol. 2026 Oct 1;17:1892694. doi: 10.3389/fneur.2026.1892694 (PMC13670917; doi:10.3389/fneur.2026.1892694)
Supplement: Supplementary file 1 [file Table_1.docx]

| **Supplementary Table S1. Top 10 journals and co-cited journals related to Lumbar disc herniation,Lumbar spinal stenosis,Cervical spondylosis,Thoracic disc herniation.** | | | | | | | | | | | |
| --- | --- | --- | --- | --- | --- | --- | --- | --- | --- | --- | --- |
| Type | Rank | Journal | Documents | Citations | IF | JCR | Rank | Co-cited journal | Citations | IF | JCR |
| Lumbar disc herniation | 1 | World Neurosurg | 296 | 3444 | 1.9 | Q3 | 1 | spine | 25786 | 2.7 | Q2 |
|  | 2 | eur spine j | 239 | 3920 | 2.6 | Q2 | 2 | eur spine j | 9428 | 2.6 | Q2 |
|  | 3 | medicine | 178 | 1295 | 1.4 | Q2 | 3 | pain physician | 6236 | 2.6 | Q2 |
|  | 4 | pain physician | 175 | 4957 | 2.6 | Q2 | 4 | spine j | 5845 | 4.9 | Q1 |
|  | 5 | spine | 169 | 3808 | 2.7 | Q2 | 5 | world neurosurg | 3769 | 1.9 | Q3 |
|  | 6 | spine j | 152 | 3232 | 4.9 | Q1 | 6 | j neurosurg-spine | 3602 | 2.9 | Q2 |
|  | 7 | bmc musculoskel dis | 148 | 1585 | 2.2 | Q2 | 7 | j bone joint surg am | 2630 | 4.4 | Q1 |
|  | 8 | cureus journal of medical science | 95 | 277 | 1.0 | Q3 | 8 | neurosurgery | 2337 | 3.9 | Q1 |
|  | 9 | global spine j | 94 | 807 | 2.6 | Q2 | 9 | clin orthop relat r | 1921 | 4.4 | Q1 |
|  | 10 | j neurosurg-spine | 92 | 1753 | 2.9 | Q2 | 10 | bmc musculoskel dis | 1823 | 2.2 | Q2 |
| Lumbar spinal stenosis | 1 | eur spine j | 329 | 4350 | 2.6 | Q2 | 1 | spine | 27924 | 2.7 | Q2 |
|  | 2 | world neurosurg | 301 | 3610 | 1.9 | Q3 | 2 | eur spine j | 10346 | 2.6 | Q2 |
|  | 3 | spine | 279 | 6405 | 2.7 | Q2 | 3 | spine j | 7325 | 4.9 | Q1 |
|  | 4 | spine j | 218 | 4547 | 4.9 | Q1 | 4 | j neurosurg-spine | 5771 | 2.9 | Q2 |
|  | 5 | bmc musculoskel dis | 167 | 1541 | 2.2 | Q2 | 5 | world neurosurg | 3600 | 1.9 | Q3 |
|  | 6 | j neurosurg-spine | 141 | 2574 | 2.9 | Q2 | 6 | pain physician | 3392 | 2.6 | Q2 |
|  | 7 | medicine | 120 | 793 | 1.4 | Q2 | 7 | j bone joint surg am | 2935 | 4.4 | Q1 |
|  | 8 | global spine j | 116 | 1555 | 2.6 | Q2 | 8 | new engl j med | 2547 | 96.3 | Q1 |
|  | 9 | cureus journal of medical science | 105 | 262 | 1.0 | Q3 | 9 | neurosurgery | 2361 | 3.9 | Q1 |
|  | 10 | clinical spine surgery | 101 | 1279 | 1.6 | Q3 | 10 | j spinal disord tech | 2011 | 2.3 | Q3 |
| Cervical spondylosis | 1 | world neurosurg | 302 | 3033 | 5.7 | Q1 | 1 | spine | 24294 | 2.7 | Q2 |
|  | 2 | spine | 284 | 5634 | 2.7 | Q2 | 2 | eur spine j | 8565 | 2.6 | Q2 |
|  | 3 | eur spine j | 208 | 3933 | 2.6 | Q2 | 3 | j neurosurg-spine | 6642 | 2.9 | Q2 |
|  | 4 | clinical spine surgery | 174 | 1457 | 2.7 | Q2 | 4 | spine j | 5633 | 4.9 | Q1 |
|  | 5 | global spine j | 163 | 2704 | 2.6 | Q1 | 5 | neurosurgery | 4152 | 3.9 | Q1 |
|  | 6 | j neurosurg-spine | 150 | 2558 | 4.7 | Q1 | 6 | world neurosurg | 2940 | 1.9 | Q3 |
|  | 7 | spine j | 137 | 1828 | 4.9 | Q1 | 7 | j bone joint surg am | 2815 | 4.4 | Q1 |
|  | 8 | medicine | 113 | 1116 | 1.6 | Q3 | 8 | glob spine j | 2555 | 2.6 | Q2 |
|  | 9 | neurosurgery | 99 | 1775 | 4.4 | Q1 | 9 | j neurosurg | 2439 | 3.5 | Q1 |
|  | 10 | journal of clinical neuroscience | 89 | 803 | 2.6 | Q2 | 10 | j spinal disord tech | 2386 | 2.3 | Q3 |
| Thoracic disc herniation | 1 | world neurosurg | 28 | 246 | 1.9 | Q3 | 1 | spine | 1804 | 2.7 | Q2 |
|  | 2 | eur spine j | 24 | 276 | 2.6 | Q2 | 2 | eur spine j | 944 | 2.6 | Q2 |
|  | 3 | spine | 15 | 167 | 2.7 | Q2 | 3 | pain physician | 803 | 2.6 | Q2 |
|  | 4 | acta neurochirurgica | 12 | 33 | 1.9 | Q3 | 4 | j neurosurg-spine | 615 | 2.9 | Q2 |
|  | 5 | global spine j | 12 | 165 | 2.6 | Q2 | 5 | spine j | 456 | 4.9 | Q1 |
|  | 6 | journal of clinical neuroscience | 11 | 100 | 1.9 | Q3 | 6 | j neurosurg | 430 | 3.5 | Q1 |
|  | 7 | operative neurosurgery | 11 | 49 | 2.2 | Q3 | 7 | world neurosurg | 354 | 1.9 | Q3 |
|  | 8 | pain physician | 11 | 623 | 2.6 | Q2 | 8 | neurosurgery | 325 | 3.9 | Q1 |
|  | 9 | british journal of neurosurgery | 10 | 31 | 1.0 | Q4 | 9 | neurosurg focus | 239 | 3.3 | Q2 |
|  | 10 | medicine | 10 | 61 | 1.4 | Q2 | 10 | j spinal disord tech | 191 | 2.3 | Q3 |
