# Supplementary material for: Age-related spinal degenerative diseases: a quantitative analysis of the dynamic evolution, knowledge structure, and research frontiers in neurological damage
Source: Front Neurol. 2026 Oct 1;17:1892694. doi: 10.3389/fneur.2026.1892694 (PMC13670917; doi:10.3389/fneur.2026.1892694)
Supplement: Supplementary file 2 [file Table_2.docx]

| **Supplementary Table S2.Top 10 references related to Lumbar disc herniation,Lumbar spinal stenosis,Cervical spondylosis,Thoracic disc herniation.** | | | | | | | |
| --- | --- | --- | --- | --- | --- | --- | --- |
| Type | Rank | Literature | Title | DOI | Source | IF/JCR | Citations |
| Lumbar disc herniation | 1 | Pfirrmann（2001） | Magnetic resonance classification of lumbar intervertebral disc degeneration (Pfirrmann et al., 2001). | https://doi.org/10.1097/00007632-200109010-00011 | Spine | 2.7/Q2 | 423 |
|  | 2 | ruetten (2008) | Full-endoscopic interlaminar and transforaminal lumbar discectomy versus conventional microsurgical technique: a prospective, randomized, controlled study (Ruetten et al., 2008). | https://doi.org/10.1097/brs.0b013e31816c8af7 | Spine | 2.7/Q2 | 350 |
|  | 3 | Yeung（2002） | Posterolateral endoscopic excision for lumbar disc herniation: Surgical technique, outcome, and complications in 307 consecutive cases (Yeung and Tsou, 2002). | https://doi.org/10.1097/00007632-200204010-00009 | Spine | 2.7/Q2 | 316 |
|  | 4 | Fairbank（2000） | The Oswestry Disability Index (Fairbank and Pynsent, 2000). | https://doi.org/10.1097/00007632-200011150-00017 | Spine | 2.7/Q2 | 304 |
|  | 5 | Kreiner（2014） | An evidence-based clinical guideline for the diagnosis and treatment of lumbar disc herniation with radiculopathy (Kreiner et al., 2014). | https://doi.org/10.1016/j.spinee.2013.08.003 | Spine J | 4.9/Q1 | 293 |
|  | 6 | Manchikanti（2014） | An update of comprehensive evidence-based guidelines for interventional techniques in chronic spinal pain. Part II: guidance and recommendations (Manchikanti et al., 2013). | https://doi.org/10.1111/ner.12018 | Pain physician | 2.6/Q2 | 268 |
|  | 7 | Weinstein（2006） | Surgical vs nonoperative treatment for lumbar disk herniation: the Spine Patient Outcomes Research Trial (SPORT) observational cohort (Weinstein et al., 2006). | https://doi.org/10.1001/jama.296.20.2451 | Jama | 63.5/Q1 | 227 |
|  | 8 | Peul（2007） | Surgery versus prolonged conservative treatment for sciatica (Peul et al., 2007). | https://doi.org/10.1056/nejmoa064039 | N Engl J Med | 96.3/Q1 | 202 |
|  | 9 | Fardon（2014） | Lumbar disc nomenclature: version 2.0: Recommendations of the combined task forces of the North American Spine Society, the American Society of Spine Radiology and the American Society of Neuroradiology (Fardon et al., 2014). | https://doi.org/10.1016/j.spinee.2014.04.022 | Spine J | 4.9/Q1 | 186 |
|  | 10 | Hoogland（2006） | Transforaminal posterolateral endoscopic discectomy with or without the combination of a low-dose chymopapain: a prospective randomized study in 280 consecutive cases (Hoogland et al., 2006). | https://doi.org/10.1097/01.brs.0000245955.22358.3a | Spine | 2.7/Q2 | 180 |
| Lumbar spinal stenosis | 1 | Deyo (2010) | Trends, major medical complications, and charges associated with surgery for lumbar spinal stenosis in older adults (Deyo et al., 2010). | https://doi.org/10.1001/jama.2010.338 | Jama | 63.5/Q1 | 481 |
|  | 2 | weinstein (2008) | Surgical versus nonsurgical therapy for lumbar spinal stenosis (Weinstein et al., 2008). | https://doi.org/10.1056/nejmoa0707136 | N Engl J Med | 96.3/Q1 | 417 |
|  | 3 | Försth(2016) | A Randomized, Controlled Trial of Fusion Surgery for Lumbar Spinal Stenosis (Försth et al., 2016). | https://doi.org/10.1056/nejmoa1513721 | N Engl J Med | 96.3/Q1 | 381 |
|  | 4 | Fairbank (2000) | The Oswestry Disability Index (Fairbank and Pynsent, 2000). | https://doi.org/10.1097/00007632-200011150-00017 | Spine | 2.7/Q2 | 373 |
|  | 5 | Schizas(2010) | Qualitative Grading of Severity of Lumbar Spinal Stenosis Based on the Morphology of the Dural Sac on Magnetic Resonance Images (Schizas et al., 2010). | https://doi.org/10.1097/brs.0b013e3181d359bd | Spine | 2.7/Q2 | 343 |
|  | 6 | Ghogawala(2016) | Laminectomy plus Fusion versus Laminectomy Alone for Lumbar Spondylolisthesis (Ghogawala et al., 2016). | https://doi.org/10.1056/nejmoa1508788 | N Engl J Med | 96.3/Q1 | 321 |
|  | 7 | Kalichman (2009) | Spinal stenosis prevalence and association with symptoms: the Framingham Study (Kalichman et al., 2009). | https://doi.org/10.1016/j.spinee.2009.03.005 | Spine J | 4.9/Q1 | 275 |
|  | 8 | Lurie (2016) | Management of lumbar spinal stenosis (Lurie and Tomkins-Lane, 2016). | https://doi.org/10.1136/bmj.h6234 | BMJ | 93.7/Q1 | 273 |
|  | 9 | Katz (2008) | Clinical practice. Lumbar spinal stenosis (Katz and Harris, 2008). | https://doi.org/10.1056/nejmcp0708097 | N Engl J Med | 96.3/Q1 | 241 |
|  | 10 | Herkowitz (1991) | Degenerative lumbar spondylolisthesis with spinal stenosis. A prospective study comparing decompression with decompression and intertransverse process arthrodesis (Herkowitz and Kurz, 1991). | https://pubmed.ncbi.nlm.nih.gov/2071615/ | J Bone Joint Surg Am | 4.4/Q1 | 224 |
| Cervical spondylosis | 1 | nouri (2015) | Degenerative Cervical Myelopathy: Epidemiology, Genetics, and Pathogenesis (Nouri et al., 2015). | https://doi.org/10.1097/brs.0000000000000913 | Spine | 2.7/Q2 | 516 |
|  | 2 | fehlings (2013) | Efficacy and safety of surgical decompression in patients with cervical spondylotic myelopathy: results of the AOSpine North America prospective multi-center study (Fehlings et al., 2013). | https://doi.org/10.2106/jbjs.l.00589 | J Bone Joint Surg Am | 4.4/Q1 | 319 |
|  | 3 | hirabayashi (1981) | Operative results and postoperative progression of ossification among patients with ossification of cervical posterior longitudinal ligament (Hirabayashi et al., 1981). | https://doi.org/10.1097/00007632-198107000-00005 | Spine | 2.7/Q2 | 282 |
|  | 4 | kalsi (2013) | Cervical spondylotic myelopathy: the clinical phenomenon and the current pathobiology of an increasingly prevalent and devastating disorder (Kalsi-Ryan et al., 2013). | https://doi.org/10.1177/1073858412467377 | Neuroscientist | 3.6/Q1 | 268 |
|  | 5 | fehlings(2017) | A Clinical Practice Guideline for the Management of Acute Spinal Cord Injury: Introduction, Rationale, and Scope (Fehlings et al., 2017). | https://doi.org/10.1177/2192568217703387 | Global Spine J | 2.6/Q2 | 264 |
|  | 6 | karadimas (2013) | Pathophysiology and natural history of cervical spondylotic myelopathy (Karadimas et al., 2013). | https://doi.org/10.1097/brs.0b013e3182a7f2c3 | Spine | 2.7/Q2 | 253 |
|  | 7 | badhiwala (2020) | Degenerative cervical myelopathy - update and future directions (Badhiwala et al., 2020). | https://doi.org/10.1038/s41582-019-0303-0 | Nat Rev Neurol | 28.2/Q1 | 237 |
|  | 8 | hilibrand (1999) | Radiculopathy and myelopathy at segments adjacent to the site of a previous anterior cervical arthrodesis (Hilibrand et al., 1999). | https://doi.org/10.2106/00004623-199904000-00009 | J Bone Joint Surg Am | 4.4/Q1 | 214 |
|  | 9 | fehlings (2015) | A global perspective on the outcomes of surgical decompression in patients with cervical spondylotic myelopathy: results from the prospective multicenter AOSpine international study on 479 patients (Fehlings et al., 2015). | https://doi.org/10.1097/brs.0000000000000988 | Spine | 2.7/Q2 | 207 |
|  | 10 | benzel(1991) | Cervical laminectomy and dentate ligament section for cervical spondylotic myelopathy (Benzel et al., 1991). | https://doi.org/10.1097/00002517-199109000-00005 | J Spinal Disord | 2.3/Q3 | 200 |
| Thoracic disc herniation | 1 | hott (2005) | Surgical management of giant herniated thoracic discs: analysis of 20 cases (Hott et al., 2005). | https://doi.org/10.3171/spi.2005.3.3.0191 | J Neurosurg Spine | 2.9/Q2 | 61 |
|  | 2 | quint (2012) | Thoracoscopic treatment for single level symptomatic thoracic disc herniation: a prospective followed cohort study in a group of 167 consecutive cases (Quint et al., 2012). | https://doi.org/10.1007/s00586-011-2103-0 | Eur Spine J | 2.6/Q2 | 58 |
|  | 3 | stillerman(1998) | Experience in the surgical management of 82 symptomatic herniated thoracic discs and review of the literature (Stillerman et al., 1998). | https://doi.org/10.3171/jns.1998.88.4.0623 | J Neurosurg | 3.5/Q1 | 53 |
|  | 4 | arts (2014) | Anterior or posterior approach of thoracic disc herniation? A comparative cohort of mini-transthoracic versus transpedicular discectomies (Arts and Bartels, 2014). | https://doi.org/10.1016/j.spinee.2013.09.053 | Spine J | 4.9/Q1 | 48 |
|  | 5 | Court(2018) | Thoracic disc herniation: Surgical treatment (Court et al., 2018). | https://doi.org/10.1016/j.otsr.2017.04.022 | Orthop Traumatol Surg Res | 2.3/Q2 | 46 |
|  | 6 | manchikanti(2014) | An update of comprehensive evidence-based guidelines for interventional techniques in chronic spinal pain. Part II: guidance and recommendations (Manchikanti et al., 2013). | https://doi.org/10.1111/ner.12018 | Pain Physician | 2.6/Q2 | 45 |
|  | 7 | bouthors(2019) | Surgical treatment of thoracic disc herniation: an overview (Bouthors et al., 2019). | https://doi.org/10.1007/s00264-018-4224-0 | Int Orthop | 2/Q2 | 38 |
|  | 8 | anand (2002) | Video-assisted thoracoscopic surgery for thoracic disc disease - Classification and outcome study of 100 consecutive cases with a 2-year minimum follow-up period (Anand and Regan, 2002). | https://doi.org/10.1097/00007632-200204150-00018 | Spine | 2.7/Q2 | 37 |
|  | 9 | gille (2017) | A new classification system for degenerative spondylolisthesis of the lumbar spine (Gille et al., 2017). | https://doi.org/10.1007/s00586-017-5275-4 | Eur Spine J | 2.6/Q2 | 37 |
|  | 10 | arce (1985) | Herniated thoracic disks (Arce and Dohrmann, 1985). | https://pubmed.ncbi.nlm.nih.gov/3894922/ | Neurol Clin | 2.4/Q2 | 34 |

Pfirrmann, C.W., Metzdorf, A., Zanetti, M., Hodler, J., Boos, N., 2001. Magnetic resonance classification of lumbar intervertebral disc degeneration. Spine (Phila Pa 1976) 26(17), 1873-1878. <https://doi.org/10.1097/00007632-200109010-00011>.

Ruetten, S., Komp, M., Merk, H., Godolias, G., 2008. Full-endoscopic interlaminar and transforaminal lumbar discectomy versus conventional microsurgical technique: a prospective, randomized, controlled study. Spine (Phila Pa 1976) 33(9), 931-939. <https://doi.org/10.1097/BRS.0b013e31816c8af7>.

Yeung, A.T., Tsou, P.M., 2002. Posterolateral endoscopic excision for lumbar disc herniation: Surgical technique, outcome, and complications in 307 consecutive cases. Spine (Phila Pa 1976) 27(7), 722-731. <https://doi.org/10.1097/00007632-200204010-00009>.

Kreiner, D.S., Hwang, S.W., Easa, J.E., Resnick, D.K., Baisden, J.L., Bess, S., Cho, C.H., DePalma, M.J., Dougherty, P., 2nd, Fernand, R., Ghiselli, G., Hanna, A.S., Lamer, T., Lisi, A.J., Mazanec, D.J., Meagher, R.J., Nucci, R.C., Patel, R.D., Sembrano, J.N., Sharma, A.K., Summers, J.T., Taleghani, C.K., Tontz, W.L., Jr., Toton, J.F., 2014. An evidence-based clinical guideline for the diagnosis and treatment of lumbar disc herniation with radiculopathy. Spine J 14(1), 180-191. <https://doi.org/10.1016/j.spinee.2013.08.003>.

Manchikanti, L., Abdi, S., Atluri, S., Benyamin, R.M., Boswell, M.V., Buenaventura, R.M., Bryce, D.A., Burks, P.A., Caraway, D.L., Calodney, A.K., Cash, K.A., Christo, P.J., Cohen, S.P., Colson, J., Conn, A., Cordner, H., Coubarous, S., Datta, S., Deer, T.R., Diwan, S., Falco, F.J., Fellows, B., Geffert, S., Grider, J.S., Gupta, S., Hameed, H., Hameed, M., Hansen, H., Helm, S., 2nd, Janata, J.W., Justiz, R., Kaye, A.D., Lee, M., Manchikanti, K.N., McManus, C.D., Onyewu, O., Parr, A.T., Patel, V.B., Racz, G.B., Sehgal, N., Sharma, M.L., Simopoulos, T.T., Singh, V., Smith, H.S., Snook, L.T., Swicegood, J.R., Vallejo, R., Ward, S.P., Wargo, B.W., Zhu, J., Hirsch, J.A., 2013. An update of comprehensive evidence-based guidelines for interventional techniques in chronic spinal pain. Part II: guidance and recommendations. Pain Phys. 16(2 Suppl), S49-283.

Weinstein, J.N., Lurie, J.D., Tosteson, T.D., Skinner, J.S., Hanscom, B., Tosteson, A.N., Herkowitz, H., Fischgrund, J., Cammisa, F.P., Albert, T., Deyo, R.A., 2006. Surgical vs nonoperative treatment for lumbar disk herniation: the Spine Patient Outcomes Research Trial (SPORT) observational cohort. JAMA 296(20), 2451-2459. <https://doi.org/10.1001/jama.296.20.2451>.

D., Hilibrand, A., Goldberg, H., Berven, S., An, H., 2008. Surgical versus nonsurgical therapy for lumbar spinal stenosis. N Engl J Med 358(8), 794-810. <https://doi.org/10.1056/NEJMoa0707136>.

Försth, P., Ólafsson, G., Carlsson, T., Frost, A., Borgström, F., Fritzell, P., Öhagen, P., Michaëlsson, K., Sandén, B., 2016. A Randomized, Controlled Trial of Fusion Surgery for Lumbar Spinal Stenosis. N Engl J Med 374(15), 1413-1423. <https://doi.org/10.1056/NEJMoa1513721>.

Fairbank, J.C., Pynsent, P.B., 2000. The Oswestry Disability Index. Spine (Phila Pa 1976) 25(22), 2940-2952; discussion 2952. <https://doi.org/10.1097/00007632-200011150-00017>.

Schizas, C., Theumann, N., Burn, A., Tansey, R., Wardlaw, D., Smith, F.W., Kulik, G., 2010. Qualitative grading of severity of lumbar spinal stenosis based on the morphology of the dural sac on magnetic resonance images. Spine (Phila Pa 1976) 35(21), 1919-1924. <https://doi.org/10.1097/BRS.0b013e3181d359bd>.

Ghogawala, Z., Dziura, J., Butler, W.E., Dai, F., Terrin, N., Magge, S.N., Coumans, J.V., Harrington, J.F., Amin-Hanjani, S., Schwartz, J.S., Sonntag, V.K., Barker, F.G., 2nd, Benzel, E.C., 2016. Laminectomy plus Fusion versus Laminectomy Alone for Lumbar Spondylolisthesis. N Engl J Med 374(15), 1424-1434. <https://doi.org/10.1056/NEJMoa1508788>.

Kalichman, L., Cole, R., Kim, D.H., Li, L., Suri, P., Guermazi, A., Hunter, D.J., 2009. Spinal stenosis prevalence and association with symptoms: the Framingham Study. Spine J 9(7), 545-550. <https://doi.org/10.1016/j.spinee.2009.03.005>.

Katz, J.N., Harris, M.B., 2008. Clinical practice. Lumbar spinal stenosis. N Engl J Med 358(8), 818-825. <https://doi.org/10.1056/NEJMcp0708097>.

Herkowitz, H.N., Kurz, L.T., 1991. Degenerative lumbar spondylolisthesis with spinal stenosis. A prospective study comparing decompression with decompression and intertransverse process arthrodesis. J Bone Joint Surg Am 73(6), 802-808.

Nouri, A., Tetreault, L., Singh, A., Karadimas, S.K., Fehlings, M.G., 2015. Degenerative Cervical Myelopathy: Epidemiology, Genetics, and Pathogenesis. Spine (Phila Pa 1976) 40(12), E675-693. <https://doi.org/10.1097/brs.0000000000000913>.

Fehlings, M.G., Wilson, J.R., Kopjar, B., Yoon, S.T., Arnold, P.M., Massicotte, E.M., Vaccaro, A.R., Brodke, D.S., Shaffrey, C.I., Smith, J.S., Woodard, E.J., Banco, R.J., Chapman, J.R., Janssen, M.E., Bono, C.M., Sasso, R.C., Dekutoski, M.B., Gokaslan, Z.L., 2013. Efficacy and safety of surgical decompression in patients with cervical spondylotic myelopathy: results of the AOSpine North America prospective multi-center study. J Bone Joint Surg Am 95(18), 1651-1658. <https://doi.org/10.2106/jbjs.L.00589>.

Hirabayashi, K., Miyakawa, J., Satomi, K., Maruyama, T., Wakano, K., 1981. Operative results and postoperative progression of ossification among patients with ossification of cervical posterior longitudinal ligament. Spine (Phila Pa 1976) 6(4), 354-364. <https://doi.org/10.1097/00007632-198107000-00005>.

Kalsi-Ryan, S., Karadimas, S.K., Fehlings, M.G., 2013. Cervical spondylotic myelopathy: the clinical phenomenon and the current pathobiology of an increasingly prevalent and devastating disorder. Neuroscientist 19(4), 409-421. <https://doi.org/10.1177/1073858412467377>.

Fehlings, M.G., Tetreault, L.A., Wilson, J.R., Kwon, B.K., Burns, A.S., Martin, A.R., Hawryluk, G., Harrop, J.S., 2017. A Clinical Practice Guideline for the Management of Acute Spinal Cord Injury: Introduction, Rationale, and Scope. Global Spine J 7(3 Suppl), 84s-94s. <https://doi.org/10.1177/2192568217703387>.

Karadimas, S.K., Erwin, W.M., Ely, C.G., Dettori, J.R., Fehlings, M.G., 2013. Pathophysiology and natural history of cervical spondylotic myelopathy. Spine (Phila Pa 1976) 38(22 Suppl 1), S21-36. <https://doi.org/10.1097/BRS.0b013e3182a7f2c3>.

Lurie, J., Tomkins-Lane, C., 2016. Management of lumbar spinal stenosis. Bmj 352, h6234. <https://doi.org/10.1136/bmj.h6234>.

Badhiwala, J.H., Ahuja, C.S., Akbar, M.A., Witiw, C.D., Nassiri, F., Furlan, J.C., Curt, A., Wilson, J.R., Fehlings, M.G., 2020. Degenerative cervical myelopathy - update and future directions. Nat Rev Neurol 16(2), 108-124. <https://doi.org/10.1038/s41582-019-0303-0>.

Hilibrand, A.S., Carlson, G.D., Palumbo, M.A., Jones, P.K., Bohlman, H.H., 1999. Radiculopathy and myelopathy at segments adjacent to the site of a previous anterior cervical arthrodesis. J Bone Joint Surg Am 81(4), 519-528. <https://doi.org/10.2106/00004623-199904000-00009>.

Fehlings, M.G., Ibrahim, A., Tetreault, L., Albanese, V., Alvarado, M., Arnold, P., Barbagallo, G., Bartels, R., Bolger, C., Defino, H., Kale, S., Massicotte, E., Moraes, O., Scerrati, M., Tan, G., Tanaka, M., Toyone, T., Yukawa, Y., Zhou, Q., Zileli, M., Kopjar, B., 2015. A global perspective on the outcomes of surgical decompression in patients with cervical spondylotic myelopathy: results from the prospective multicenter AOSpine international study on 479 patients. Spine (Phila Pa 1976) 40(17), 1322-1328. <https://doi.org/10.1097/brs.0000000000000988>.

Benzel, E.C., Lancon, J., Kesterson, L., Hadden, T., 1991. Cervical laminectomy and dentate ligament section for cervical spondylotic myelopathy. J Spinal Disord 4(3), 286-295. <https://doi.org/10.1097/00002517-199109000-00005>.

.Hott, J.S., Feiz-Erfan, I., Kenny, K., Dickman, C.A., 2005. Surgical management of giant herniated thoracic discs: analysis of 20 cases. J Neurosurg Spine 3(3), 191-197. <https://doi.org/10.3171/spi.2005.3.3.0191>.

Quint, U., Bordon, G., Preissl, I., Sanner, C., Rosenthal, D., 2012. Thoracoscopic treatment for single level symptomatic thoracic disc herniation: a prospective followed cohort study in a group of 167 consecutive cases. Eur Spine J 21(4), 637-645. <https://doi.org/10.1007/s00586-011-2103-0>.

Stillerman, C.B., Chen, T.C., Couldwell, W.T., Zhang, W., Weiss, M.H., 1998. Experience in the surgical management of 82 symptomatic herniated thoracic discs and review of the literature. J Neurosurg 88(4), 623-633. <https://doi.org/10.3171/jns.1998.88.4.0623>.

Arts, M.P., Bartels, R.H., 2014. Anterior or posterior approach of thoracic disc herniation? A comparative cohort of mini-transthoracic versus transpedicular discectomies. Spine J 14(8), 1654-1662. <https://doi.org/10.1016/j.spinee.2013.09.053>.

Manchikanti, L., Abdi, S., Atluri, S., Benyamin, R.M., Boswell, M.V., Buenaventura, R.M., Bryce, D.A., Burks, P.A., Caraway, D.L., Calodney, A.K., Cash, K.A., Christo, P.J., Cohen, S.P., Colson, J., Conn, A., Cordner, H., Coubarous, S., Datta, S., Deer, T.R., Diwan, S., Falco, F.J., Fellows, B., Geffert, S., Grider, J.S., Gupta, S., Hameed, H., Hameed, M., Hansen, H., Helm, S., 2nd, Janata, J.W., Justiz, R., Kaye, A.D., Lee, M., Manchikanti, K.N., McManus, C.D., Onyewu, O., Parr, A.T., Patel, V.B., Racz, G.B., Sehgal, N., Sharma, M.L., Simopoulos, T.T., Singh, V., Smith, H.S., Snook, L.T., Swicegood, J.R., Vallejo, R., Ward, S.P., Wargo, B.W., Zhu, J., Hirsch, J.A., 2013. An update of comprehensive evidence-based guidelines for interventional techniques in chronic spinal pain. Part II: guidance and recommendations. Pain Phys. 16(2 Suppl), S49-283.

Bouthors, C., Benzakour, A., Court, C., 2019. Surgical treatment of thoracic disc herniation: an overview. Int Orthop 43(4), 807-816. <https://doi.org/10.1007/s00264-018-4224-0>.

Gille, O., Bouloussa, H., Mazas, S., Vergari, C., Challier, V., Vital, J.M., Coudert, P., Ghailane, S., 2017. A new classification system for degenerative spondylolisthesis of the lumbar spine. Eur Spine J 26(12), 3096-3105. <https://doi.org/10.1007/s00586-017-5275-4>.

Arce, C.A., Dohrmann, G.J., 1985. Herniated thoracic disks. Neurol Clin 3(2), 383-392.

Fairbank JC, Pynsent PB. The Oswestry Disability Index. Spine (Phila Pa 1976). 2000;25(22):2940-2952. doi:10.1097/00007632-200011150-00017

Peul WC, van Houwelingen HC, van den Hout WB, Brand R, Eekhof JA, Tans JT, et al. Surgery versus prolonged conservative treatment for sciatica. N Engl J Med. 2007;356(22):2245-2256. doi:10.1056/NEJMoa064039

Deyo RA, Mirza SK, Martin BI, Kreuter W, Goodman DC, Jarvik JG. Trends, major medical complications, and charges associated with surgery for lumbar spinal stenosis in older adults. JAMA. 2010;303(13):1259-1265. doi:10.1001/jama.2010.338

Court C, Mansour E, Bouthors C. Thoracic disc herniation: Surgical treatment. Orthop Traumatol Surg Res. 2018;104(1S):S31-S40. doi:10.1016/j.otsr.2017.04.022

Anand N, Regan JJ. Video-assisted thoracoscopic surgery for thoracic disc disease: Classification and outcome study of 100 consecutive cases with a 2-year minimum follow-up period. Spine (Phila Pa 1976). 2002;27(8):871-879. doi:10.1097/00007632-200204150-00018

Fardon, D.F., Williams, A.L., Dohring, E.J., Murtagh, F.R., Gabriel Rothman, S.L., Sze, G.K., 2014. Lumbar disc nomenclature: version 2.0: Recommendations of the combined task forces of the North American Spine Society, the American Society of Spine Radiology and the American Society of Neuroradiology. Spine J 14(11), 2525-2545. <https://doi.org/10.1016/j.spinee.2014.04.022>.

Hoogland, T., Schubert, M., Miklitz, B., Ramirez, A., 2006. Transforaminal posterolateral endoscopic discectomy with or without the combination of a low-dose chymopapain: a prospective randomized study in 280 consecutive cases. Spine (Phila Pa 1976) 31(24), E890-897. <https://doi.org/10.1097/01.brs.0000245955.22358.3a>.
