# Supplementary material for: Age-related spinal degenerative diseases: a quantitative analysis of the dynamic evolution, knowledge structure, and research frontiers in neurological damage
Source: Front Neurol. 2026 Oct 1;17:1892694. doi: 10.3389/fneur.2026.1892694 (PMC13670917; doi:10.3389/fneur.2026.1892694)
Supplement: Supplementary file 3 [file Table_3.DOCX]

| Search Strategy | | | |
| --- | --- | --- | --- |
| **Tape** | **WOSCC** | **Scopus** | **Pubmed** |
| lumbar disc herniation | (((((((TS=(lumbar disc herniation)) OR TS=(lumbar disc protrusion)) OR TS=(lumbar intervertebral disc herniation )) OR TS=(lumbar intervertebral disc prolapse)) OR TS=(lumbar herniated disc lumbar disc protrusion)) OR TS=(lumbar intervertebral disc herniation )) OR TS=(prolapse of lumbar intervertebral disc )) OR TS=(protrusion of lumbar vertebral disc ) | (TITLE-ABS-KEY (lumbar AND disc AND herniation) OR TITLE-ABS-KEY (lumbar AND disc AND protrusion) OR TITLE-ABS-KEY (lumbar AND intervertebral AND disc AND herniation) OR TITLE-ABS-KEY (lumbar AND intervertebral AND disc AND prolapse) OR TITLE-ABS-KEY (lumbar AND herniated AND disc AND lumbar AND disc AND protrusion) OR TITLE-ABS-KEY (lumbar AND intervertebral AND disc AND herniation) OR TITLE-ABS-KEY (prolapse AND of AND lumbar AND intervertebral AND disc) OR TITLE-ABS-KEY (protrusion AND of AND lumbar AND vertebral AND disc) ) AND PUBYEAR > 2014 AND PUBYEAR < 2026 | ((((((((lumbar disc herniation[Title/Abstract]) OR (LDH[Title/Abstract])) OR (lumbar disc protrusion[Title/Abstract])) OR (lumbar intervertebral disc herniation[Title/Abstract])) OR (lumbar intervertebral disc prolapse[Title/Abstract])) OR (lumbar herniated disc lumbar disc protrusion[Title/Abstract])) OR (lumbar intervertebral disc herniation[Title/Abstract])) OR (prolapse of lumbar intervertebral disc[Title/Abstract])) OR (protrusion of lumbar vertebral disc[Title/Abstract]) |
| cervical spondylosis | (((((TS=(cervical spondylosis)) OR TS=(cervical spondylopathy)) OR ALL=(cervical spondylotic myelopathy))) OR ALL=(Degenerative Cervical spondylosis)) OR ALL=(Degenerative Cervical Myelopathy) | ( TITLE-ABS-KEY ( cervical AND spondylosis ) OR TITLE-ABS-KEY ( cervical AND spondylopathy ) OR TITLE-ABS-KEY ( cervical AND spondylotic AND myelopathy ) OR TITLE-ABS-KEY ( degenerative AND cervical AND spondylosis ) OR TITLE-ABS-KEY ( degenerative AND cervical AND myelopathy ) ) AND PUBYEAR > 2014 AND PUBYEAR < 2026 | ((((cervical spondylosis[Title/Abstract]) OR (cervical spondylopathy[Title/Abstract])) OR (cervical spondylotic myelopathy[Title/Abstract])) OR (Degenerative Cervical spondylosis[Title/Abstract])) OR (Degenerative Cervical Myelopathy[Title/Abstract]) |
| Thoracic disc herniation | (TS=(Thoracic disc herniation)) OR TS=(thoracic disc protrusion) | ( TITLE-ABS-KEY ( thoracic AND disc AND herniation ) AND TITLE-ABS-KEY ( thoracic AND disc AND protrusion ) ) AND PUBYEAR > 2014 AND PUBYEAR < 2026 | (thoracic disc protrusion[Title/Abstract]) OR (Thoracic disc herniation[Title/Abstract]) |
| lumbar spinal stenosis | TS=(lumbar spinal stenosis) | TITLE-ABS-KEY ( lumbar AND spinal AND stenosis ) AND PUBYEAR > 2014 AND PUBYEAR < 2026 | lumbar spinal stenosis[Title/Abstract] |
